# Distinct Z-DNA binding mode of a PKR-like protein kinase containing a Z-DNA binding domain (PKZ)

**DOI:** 10.1093/nar/gku189

**Published:** 2014-03-20

**Authors:** Doyoun Kim, Jeonghwan Hur, Kwangsoo Park, Sangsu Bae, Donghyuk Shin, Sung Chul Ha, Hye-Yeon Hwang, Sungchul Hohng, Joon-Hwa Lee, Sangho Lee, Yang-Gyun Kim, Kyeong Kyu Kim

**Affiliations:** 1 Department of Molecular Cell Biology, Samsung Biomedical Research Institute, Sungkyunkwan University School of Medicine, Suwon 440-746, Korea; 2 Department of Physics and Astronomy, Seoul National University, Seoul 151-747, Korea; 3 National Center for Creative Research Initiatives, Seoul National University, Seoul 151-747, Korea; 4 Department of Biological Sciences, Sungkyunkwan University, Suwon 440-746, Korea; 5 Pohang Accelerator Laboratory, Pohang University of Science and Technology, Pohang, Kyungbuk 790-784, Korea; 6 Department of Biophysics and Chemical Biology, Seoul National University, Seoul 151-747, Korea; 7 Department of Chemistry and RINS, Gyeongsang National University, Jinju, Gyeongnam 660-701, Korea; 8 Department of Chemistry, Sungkyunkwan University, Suwon 440-746, Korea

## Abstract

Double-stranded ribonucleic acid-activated protein kinase (PKR) downregulates translation as a defense mechanism against viral infection. In fish species, PKZ, a PKR-like protein kinase containing left-handed deoxyribonucleic acid (Z-DNA) binding domains, performs a similar role in the antiviral response. To understand the role of PKZ in Z-DNA recognition and innate immune response, we performed structural and functional studies of the Z-DNA binding domain (Zα) of PKZ from *Carassius auratus* (caZα_PKZ_). The 1.7-Å resolution crystal structure of caZα_PKZ_:Z-DNA revealed that caZα_PKZ_ shares the overall fold with other Zα, but has discrete structural features that differentiate its DNA binding mode from others. Functional analyses of caZα_PKZ_ and its mutants revealed that caZα_PKZ_ mediates the fastest B-to-Z transition of DNA among Zα, and the minimal interaction for Z-DNA recognition is mediated by three backbone phosphates and six residues of caZα_PKZ_. Structure-based mutagenesis and B-to-Z transition assays confirmed that Lys56 located in the β-wing contributes to its fast B-to-Z transition kinetics. Investigation of the DNA binding kinetics of caZα_PKZ_ further revealed that the B-to-Z transition rate is positively correlated with the association rate constant. Taking these results together, we conclude that the positive charge in the β-wing largely affects fast B-to-Z transition activity by enhancing the DNA binding rate.

## INTRODUCTION

The regulation of protein synthesis is one of the most important events under stress conditions. The initiation of translation is tightly regulated as the rate limiting phase via the phosphorylation of eukaryotic initiation factor 2α (eIF2α) (1) by various protein kinases including hemeregulated inhibitor kinase, ribonucleic acid (RNA)-dependent protein kinase (PKR), PKR-like endoplasmic reticulum kinase and general control nonrepressed 2 (2,3). These kinases sense different cellular stress signals such as heme depletion, viral infection, Endoplasmic reticulum (ER) stresses and amino acid starvation, and ultimately inhibit protein synthesis and proliferation (2). Among these kinases, PKR plays an important role in the innate immune response against viral infections by recognizing double-stranded RNA (dsRNA) in the cytosol. PKR has a dsRNA binding domain (dsRBD), which consists of two dsRNA binding motifs, and an eIF2α kinase domain at the N- and C-termini, respectively (4). The binding of dsRNA to dsRBD induces the dimerization and autophosphorylation of PKR (5,6), and the activated PKR leads to apoptosis, inflammation and inhibited translation (4).

Functional orthologs of PKR have been identified in several fish species such as *Gobiocypris rarus*, *Salmo salar*, *Danio rerio*, and *Carassius auratus* (goldfish) (7–10). Since these orthologs contain two left-handed deoxyribonucleic acid (Z-DNA) binding domains (ZBDs) instead of dsRBD of PKR, they were named as protein kinases containing ZBDs (PKZ). Although the sensor domains of PKZ and PKR are different, their kinase domains are well conserved (8). Similar to PKR, PKZ has been known to reduce the overall translational level by phosphorylating Ser51 of eIF2α when it is activated by Z-DNA binding (8). Therefore, PKZ is a functional ortholog of PKR but it recognizes Z-DNA instead of dsRNA.

Left-handed Z-DNA is a higher energy conformation of the double-stranded DNA helix. Unlike canonical right-handed B-DNA, which is common in biological DNAs, Z-DNA adopts the zigzag arrangement of the phosphate backbone as a consequence of alternating *anti* and *syn* conformations of glycosidic bonds (11). Thus, alternating purine and pyrimidine sequences energetically favor the Z-DNA formation (12). Z-DNA can be stabilized by reducing the electrostatic repulsion between phosphate groups in the backbone by high ionic strength, positively charged chemicals or modification on bases (13,14). Non-aqueous solvents such as ethanol, methanol and ethylene glycol also stabilize Z-DNA (15). Besides those physicochemical factors, negative supercoiling and Z-DNA binding proteins (ZBPs) induce Z-DNA under physiological conditions (16,17), and the *in vivo* presence of Z-DNA has been demonstrated by Z-DNA-specific antibodies or ZBPs. So far, four ZBPs have been identified, each of which contains one or two ZBDs (Zα): adenosine deaminase acting on RNA-1 (ADAR1), DNA-dependent activator of interferon (IFN) regulatory factors (DAI, also known as DLM-1, and ZBP-1), protein kinase containing ZBD from fish (PKZ) and viral protein E3L (9,18–20).

Interestingly, all known ZBPs, ADAR1, DAI, E3L and PKZ, are involved in the innate immune response (17,21). Mammalian DAI recognizes cytosolic foreign DNA and consequently activates the inflammatory signal pathway through IFN and NF–κB signaling (22,23). The overexpression of ADAR1 or E3L suppresses the DNA-mediated production of IFN in mouse embryonic fibroblast cells (24). Hence, the competition between ADAR1 and DAI for cytosolic DNA has been suggested as a regulatory mechanism of the innate immune response. However, the distribution of DAI and PKZ is known to vary in different species; that is, fish have only PKZ, while mammals have only DAI, suggesting that PKZ and DAI may perform similar roles as cytosolic DNA sensors.

To date, four crystal structures of ZBDs in complex with Z-DNA are available: Zα domain of human ADAR1 (Zα_ADAR1_) (25), Zα domain of mouse DAI (Zα_DAI_) (19), Zα domain of Yaba virus (yabZα_E3L_) (26) and Zβ domain of human DAI (hZβ_DAI_) (27). Zα domains commonly have a winged helix-turn-helix (wHTH) DNA binding motif and recognize Z-DNA in a conformation-specific manner. Accordingly, most of the interactions involve backbone phosphates (17). Despite the similarity in overall structure, the binding modes and activities of Zα domains are quite distinctive (17). For example, it was proposed that the differential Z-DNA binding activities of viral Zα domains are correlated with pathogenicity (28).

**Figure 1.**
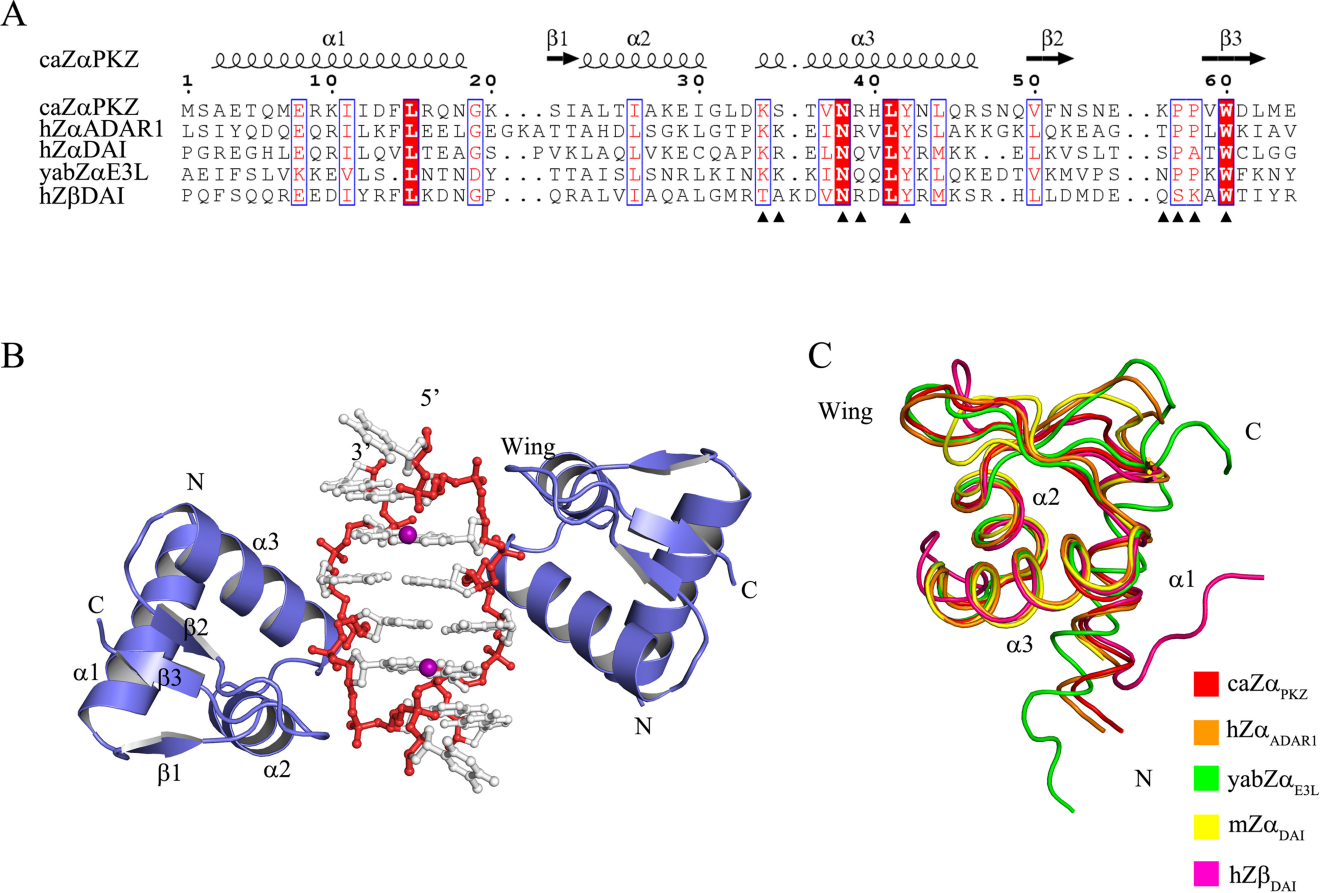
Overall structure of caZα_PKZ_ in complex with ds-dT(dCdG)_3_ and comparison with other Zα domains. (**A**) Sequence alignment of caZα_PKZ_ with human ADAR1 (hZα_ADAR1_), human DAI (hZα_DAI_) and yaba poxvirus E3L (yabZα_E3L_), and Zβ domain of human DAI (hZβ_DAI_). The secondary structures of caZα_PKZ_ are drawn at the top of the sequences. The residues interacting with the Z-DNA backbone are marked with filled triangles. (**B**) Overall structure of the caZα_PKZ_:Z-DNA complex. Double-stranded DNA and two Zα proteins are generated by C2 crystallographic symmetry. Proteins are colored slated blue, and backbones and bases of DNA are colored red and gray, respectively. The manganese ions are shown as purple spheres. (**C**) Structural comparisons of the Cα traces of caZα_PKZ_ and other Zα domains. Protein structures of caZα_PKZ_, hZα_ADAR1_ (PDB ID: 1QBJ), yabZα_E3L_ (PDB ID: 1SFU), mZα_DAI_ (PDB ID: 1J75) and hZβ_DAI_ (PDB ID: 3EYI) are drawn in red, orange, green, yellow and magenta, respectively.

caZα_PKZ_ has limited sequence identity with other Zα domains, 28% for hZα_ADAR1_, 20% for hZα_DAI_, 23% for hZβ_DAI_ and 22% for yabZα_E3L_, respectively. However, the residues involved in DNA backbone recognition are relatively well conserved with few exceptions (Figure 1) (9). It has already been proven that caZα_PKZ_ binds to double-stranded (ds) deoxy-hexanucleotide with three repeats of cytosine-guanine deoxy-dinucleotide (dCdG) in negatively supercoiled DNA and converts ds-(dCdG)_3_ from B-DNA to Z-DNA *in vitro* (29,30), but little is known about the detailed Z-DNA binding mode of caZα_PKZ_. Therefore, structural studies on Z-DNA recognition are essential to understand its binding mode to Z-DNA and its relevance to the biological function of caZα_PKZ_. In the present study, we determined the crystal structure of caZα_PKZ_ complexed with Z-DNA at 1.7-Å resolution and observed that caZα_PKZ_ mediates B-to-Z transition with the fastest kinetics among the known Zα domains. Structural analyses combined with the functional assay of caZα_PKZ_ mutants provided a basis for its Z-DNA binding mode and higher rate of B-to-Z transition.

## MATERIALS AND METHODS

### Protein preparation

The Zα domains of human DAI (hZα_DAI_), human ADAR1 (hZα_ADAR1_), E3L homolog of the yaba-like disease virus (yabZα_E3L_) and *C. auratus* PKZ (caZα_PKZ_) were prepared as reported previously (19,25,26,30). The detailed procedures are described in the Supplementary data.

### Preparation of duplex oligonucleotide

Deoxyoligonucleotides 5′-dTdCdGdCdGdCdG-3′ [dT(dCdG)_3_] and 5′-dCdGdCdGdCdGdCdGdCdGdCdG-3′ [(dCdG)_6_] were purchased from Integrated DNA Technologies (IDT, CA, USA). All oligonucleotides were in 50-mM Tris-HCl pH 8.0, 50-mM NaCl and 1-mM ethylenediaminetetraacetic acid. Duplex deoxyoligonucleotides were prepared by annealing an equimolar mixture of complementary sequences and dialyzing against buffer A. After purification using a MonoQ column (GE Healthcare, NJ, USA), the concentration of the duplex DNA was calculated spectroscopically.

### Circular dichroism measurement

The B-to-Z transition was monitored by the circular dichroism (CD) spectrum at 25°C. For each measurement, 7.5 μM of ds-(dCdG)_6_ in buffer A was used. Wild-type or mutant caZα_PKZ_ was incubated with ds-(dCdG)_6_ at final concentrations of 0 μM (0×), 7.5 μM (1×), 15 μM (2×), 30 μM (4×), 45 μM (6×) and 60 μM (8×) at 25°C for 1 h. CD spectra were recorded using a Jasco J-810 CD spectrometer between 230 nm and 320 nm at 1-nm intervals averaged over 2 s. The kinetics of the B-to-Z DNA transition of caZα_PKZ_, hZα_ADAR1_, hZα_DAI_, yabZα_E3L_ and their mutants were monitored by the time course recording of CD spectra at 255 nm for 2000 s in the presence of 15-μM ds-(dCdG)_6_.

### Structure determination

In our previous study, we confirmed that caZα_PKZ_ induces the B-to-Z transition of the ds-dT(dCdG)_3_ at 2:1 (protein:DNA) molar ratio and it forms a stable complex with Z-DNA at the same molar ratio (30). We crystallized the caZα_PKZ_:Z-DNA complex in the C2 space group using 30% PEG 1500 and 15-mM MnCl_2_ and collected diffraction data at 1.7-Å resolution. The structure of caZα_PKZ_ in complex with Z-DNA was determined by the molecular replacement method. The initial solution was obtained by *MOLREP* (32) using hZα_ADAR1_ complexed with ds-dT(dCdG)_3_ (PDB ID: 1QBJ) as a search model. Rigid body refinement of the initial solution and density modification were performed using *REFMAC* (33) and *DM* (34), respectively. Iterative model building was done using *COOT* (35). The final refinement statistics are given in Table 1. The quality of the structure was checked using MolProbity (36). All structural figures were generated using *PYMOL* (37).

**Table 1.** Data collection and refinement statistics of caZα_PKZ_/Z-DNA complex

	Native
Unit cell parameters	
Space group	C2
Unit cell	*a* = 55.37 Å
	*b* = 49.47 Å
	*c* = 29.58 Å
	β = 97.22º
Data collection	
Beamline	BL4A, PAL
Resolution range (Å)	50.0–1.70 (1.73–1.70)^a^
Redundancy	3.4 (3.0)
Completeness (%)	97.5 (89.9)^a^
*R*_merge_ (%)^b^	6.1 (32.5)^a^
*I/σ* (*I*)	43.3 (4.1)^a^
Refinement	
Resolution range (Å)	27.5–1.70 (1.94–1.70)
Number of reflections working sets	8131 (771)
Number of reflections test sets	382 (33)
Number of protein atoms	504
Number of DNA atoms	139
Number of waters	70
Number of metal ions (manganese)	1
*R*_work_/*R*_free_ (%)^c^	17.2/22.6 (19.2/25.6)
RMSD bond lengths/angles (Å)	0.022/2.226
Average B-factor (Å^2^)	25.3(protein)/21.9(DNA)/32.7(water)
Ramachandran plot (%)^d^	100.0/0.0/0.0

^a^The values in the parentheses of the resolution range, completeness, *R*_merge_ and *I*/*σ*(*I*) correspond to the last shell.

^b^*R*_merge_(*I*) = ∑_hkl_∑_j_|[*I*(*hkl*)_j_−*I*(*hkl*)]|/[∑_hkl_*I*_*hkl*_] is the jth measurement of the intensity of reflection hkl and <*I*(hkl)> is the averaged intensity.

^c^*R* = ∑_hkl_∥*F*_obs_|−|*F*_calc_∥/∑_hkl_|*F*_obs_|, where *R*_free_ is calculated without an s cutoff for a randomly chosen 5% of reflections, which were not used for structure refinement, and *R*_work_ is calculated for the remaining reflections.

^d^Percentage of residues in favored/allowed/outlier regions calculated by MolProbity (36).

## RESULTS

### Overall structure of caZα_PKZ_ in complex with Z-DNA

To understand the difference in the B-to-Z transition rates of Zα domains in relation to their Z-DNA binding modes, we determined the crystal structure of caZα_PKZ_ in complex with dsdT(dCdG)_3_ oligonucleotide at 1.7-Å resolution with *R* factor of 17.2% and *R*_free_ of 22.6%. One caZα_PKZ_ molecule bound to a single-stranded DNA was modeled in an asymmetric unit. The current model comprises 62 residues (from Ser2 to Met63) of caZα_PKZ_, seven nucleotides of single-stranded dT(dCdG)_3_ [ss-dT(dCdG)_3_] and one manganese ion. By the C2 symmetry operation, two caZα_PKZ_ molecules bound to both sides of the Z-DNA in a head-to-tail orientation were generated (Figure 1B).

The overall structure of caZα_PKZ_ is well conserved with three β strands and three α helices, like other members of the Zα family (Figure 1). With a wing between the β2 and β3 (β-wing), caZα_PKZ_ adopts the canonical wHTH structure (Figure 1B). Structure comparison revealed that caZα_PKZ_ is a homolog of other known ZBDs: the root-mean-square deviations (RMSDs) from hZα_ADAR1_ (PDB ID: 1QBJ) (25), mZα_DAI_ (PDB ID: 1J75) (19), yabZα_E3L_ (PDB ID: 1SFU) (26) and hZβ_DAI_ (PDB ID: 3EYI) (27) are 0.75 Å, 1.57 Å, 1.23 Å and 0.94 Å for 56 Cα atoms, respectively (Figure 1C). The RMSD versus residue plot also indicates that caZα_PKZ_ and hZα_ADAR1_ have no noticeable change throughout the modeled residues. For mZα_DAI_ or yabZα_E3L_, structural deviations from caZα_PKZ_ were mainly observed near the α1 helix (Figure 1C and Supplementary Figure S1). In contrast, caZα_PKZ_ and hZβ_DAI_ showed a noticeable difference in the N-terminal half of α3. The β-wing structure of caZα_PKZ_ is also largely different from that of mZα_DAI_ and hZβ_DAI_ (Supplementary Figure S1B). Structural heterogeneity in the α1 helix is expected since this helix is least conserved in sequential and structural aspects. The structural deviation in the α3 helix and the β-wing is directly related to diversity of Z-DNA binding mode since both regions have a major role in recognizing Z-DNA. For example, the conformation movement of α3 helix to 3 (10) helix has been reported in the crystal and nuclear magnetic resonance structures of hZβ_DAI_ (27,38).

In the complex structure, dsDNA molecules generated by the C2 symmetry operation adopt a canonical left-handed Z-DNA conformation (Supplementary Table S1). Like other Zα:Z-DNA complexes, all three guanines have the *syn* conformation, and the three cytosines have the *anti* conformation. Guanosines 2 and 6 (G2 and G6) adopt 4’-exo sugar pucker instead of 3′-endo sugar pucker, as frequently observed for guanine sugars in Z-DNA (Supplementary Table S2). All seven nucleotides of ds-dT(dCdG)_3_ including 5′ deoxythymidine (T0) were modeled (Supplementary Figure S2A), whereas the 5′-dT overhang was not modeled due to weak electron density in the structures of other Zα:ds-dT(dCdG)_3_ complexes. Interestingly, one manganese ion is coordinated with the backbone phosphate (P0), N7 of guanine 2 (G2) and four water molecules with an octahedral coordination geometry (Supplementary Figure S2B).

### caZα_PKZ_ has a unique Z-DNA binding mode

**Figure 2.**
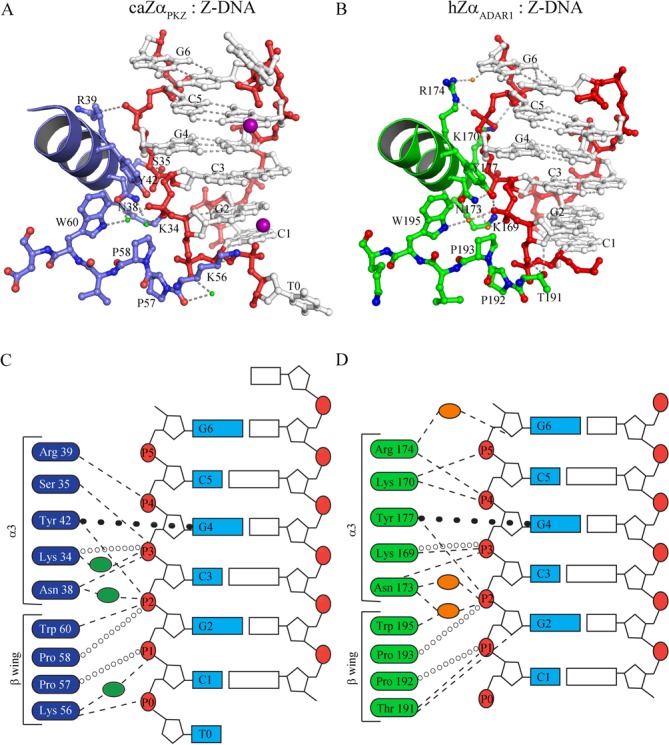
The Z-DNA binding modes of caZα_PKZ_ and hZα_ADAR1_. (**A**) The binding interface between caZα_PKZ_ and Z-DNA. The DNA binding interface of protein is depicted as a blue ribbon and ball-and-stick models. The residues involved in Z-DNA binding are labeled. The backbone and bases of DNA are drawn as red and gray ball-and-stick models. The manganese atoms are represented by magenta spheres. The water molecules are represented by green dots. (**B**) The Z-DNA binding interface of hZα_ADAR1_. The DNA binding interface of protein is depicted by a green ribbon and ball-and-stick models. The residues involved in Z-DNA binding are labeled. The backbone and bases of DNA are drawn as red and gray ball-and-stick models. The water molecules are represented by orange dots. The schematic drawings of the DNA binding interfaces of caZα_PKZ_ (**C**) and hZα_ADAR1_ (**D**) with Z-DNA. The interaction residues of caZα_PKZ_ and hZα_ADAR1_ are represented in blue (C) and green boxes (D), respectively. The phosphate backbones are numbered from P0 to P5 in red circles. Hydrogen bonds, hydrophobic interactions and π–π interaction are represented by dashed lines, open circles and closed circles, respectively. Green (C) and orange (D) ovals stand for water molecules in the caZα_PKZ_ and hZα_ADAR1_ structures, respectively.

The interaction between caZα_PKZ_ and Z-DNA is mediated by five residues in the α3 core and four residues in the β-wing. Among them, Lys34, Asn38 and Arg39 in the α3 core form direct or water-mediated hydrogen bonds with backbone phosphates of Z-DNA, as observed in the hZα_ADAR1_ structure (25) (Figures 1 and 2). Tyr42, which is known as the most critical residue for Z-DNA binding by forming the CH−π stacking with the guanine 4 (G4), is conserved in structural and sequential aspects (Figures 1A and 2B). It is interesting that caZα_PKZ_ has Ser35 in the α3 core, unlike the positively charged Lys or Arg in other Zα domains, which can form electrostatic interaction with the fifth backbone phosphate (P5; Figures 1A and 2). Due to the shorter length and lack of positive charge, Ser35 does not form ionic interactions (Figure 2A and C), but does have hydrogen bonding with the P3 phosphate. The β-wing of caZα_PKZ_ is composed of Lys56, Pro57, Pro58 and Trp60. As in other Zα domains, Trp60 forms a hydrophobic interaction with Tyr42 (19,25,26). The two proline residues, Pro57 and Pro58, interact with P1 and P2 phosphates via hydrophobic interactions. It is notable that caZα_PKZ_ has Lys56, whereas other mammalian Zα domains have a polar residue like Ser or Thr at the corresponding position. This substitution appears to be associated with the unique interaction mode of caZα_PKZ_, that is, Lys56 interacts not only with P1 but also with P0 (Figure 2A and C), thereby stabilizing T0 and Mn^2+^ ion (Supplementary Figure S2B). Accordingly, the comparison of charge distribution surfaces of Zα domains reveals that only caZα_PKZ_ has positively charged wing structure (Supplementary Figure S3). Current structural interpretations imply that the Z-DNA binding mode of caZα_PKZ_ is distinguished from those of other Zα domains, which is due to Ser35 and Lys56. Consequently, caZα_PKZ_ recognizes P0–P4 phosphates of Z-DNA, while other Zα domains interact with P1–P5 phosphates (Figure 2).

### The minimal recognition motif of caZα_PKZ_ for recognizing Z-DNA

**Figure 3.**
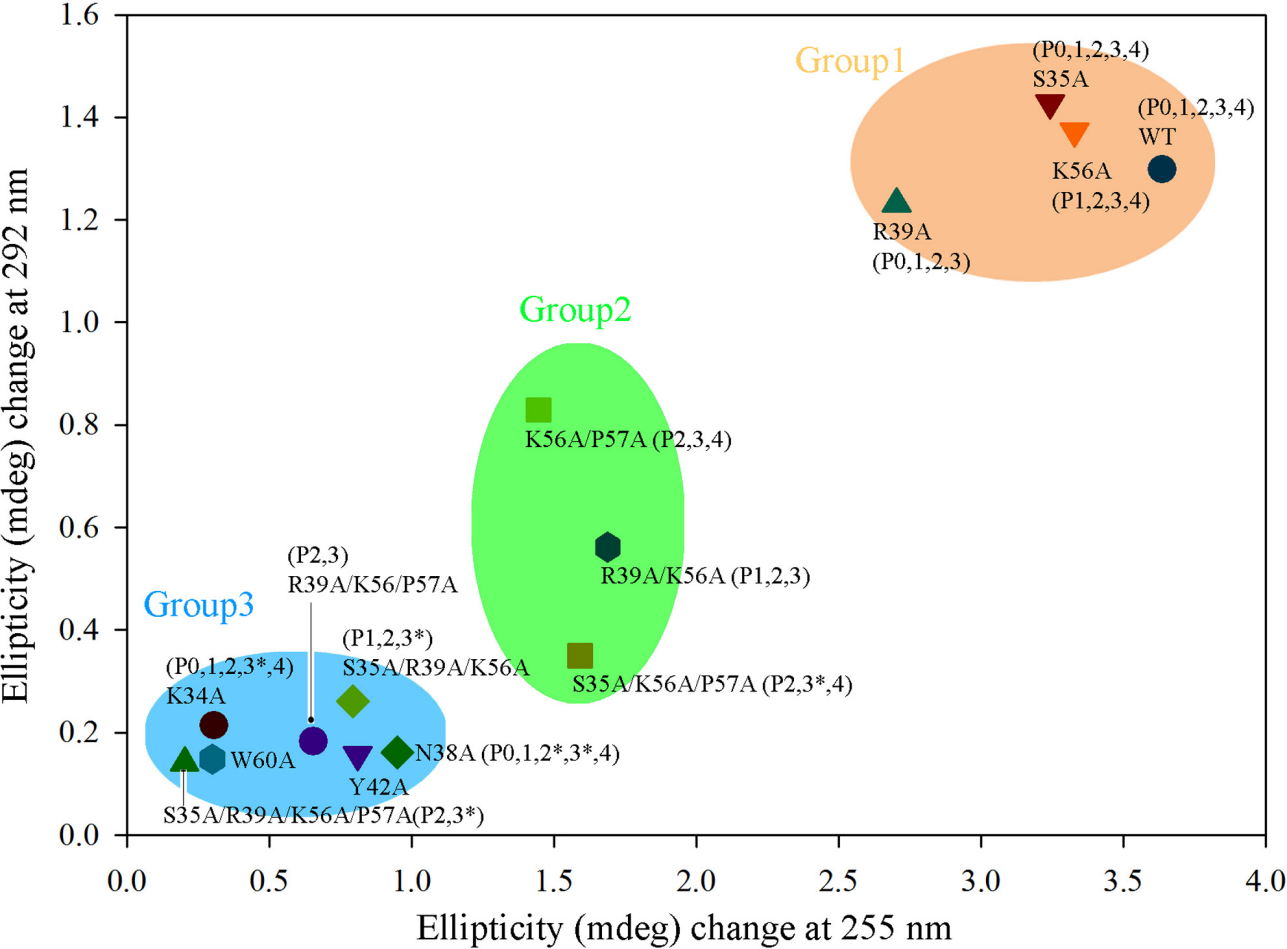
B-to-Z transition induced by caZα_PKZ_ mutants. The Y-axis and X-axis display the ellipticity (mdeg) difference during B-to-Z transition at 292 nm and 255 nm, respectively. The wild-type, K34A, S35A, N38A, R39A, Y42A, K56A, W60A, R39A/K56A, S35A/R39A/K56A, K56A/P57A, S35A/K56A/P57A, R39A/K56A/P57A and S35A/R39A/K56A/P57A mutants of caZα_PKZ_ were tested at a [P]/[N] ratio of 4. The plots are clustered in three groups by using k-means clustering algorithm. The names of phosphate groups inside of parentheses indicate the possible interaction between the residues in the mutants and the backbone phosphates. An asterisk indicates the phosphate groups that lose one of their residue interactions by mutation. In this case, their interactions with proteins partially remain.

It was proposed from the crystal structures of hZα_ADAR1_ in complex with non-CG repeat of Z-DNA that most conserved interactions are mediated by three backbone phosphates, P1, P2 and P3, and five conserved residues, Asn173, Tyr177, Pro193, Pro192 and Trp195 (39). It is clear that caZα_PKZ_ seemingly has a different binding mode to interact with Z-DNA. To elucidate the role of each residue in the DNA binding surface and to identify the minimal DNA recognition motif in caZα_PKZ_, we mutated the key interacting residues and monitored the B-to-Z transition activity of mutants at various molar ratios of protein/DNA ([P]/[N] ratios) (Supplementary Figure S4). Far-ultraviolet CD spectra (190–240 nm) confirmed that the overall folding of each caZα_PKZ_ mutant was not altered, except for Y42A and W60A mutants (Supplementary Figure S5). Since CD changes at both 255 nm and 292 nm mostly represent the B-to-Z transition, we plotted the ellipticity of DNA at 255 nm and 292 nm in the presence of mutant proteins with various [P]/[N] ratios (0–8) (Supplementary Figure S6) to compare the B-to-Z transition activity of each mutant. CD spectra showed that four molar excess of caZα_PKZ_ can fully convert ds-(dCdG)_6_ to Z-DNA, and thus the stoichiometry between caZα_PKZ_ and the ds-(dCdG)_6_ is estimated to be 4:1 (Supplementary Figure S4A). Therefore, to simplify the comparison, the ellipticity changes at 255 and 292 nm from those of B-DNA at the [P]/[N] ratio of 4 were plotted for each mutant (Figure 3). In addition, we introduced the names of backbone phosphates, which are involved in the binding to each mutant in the same plot. Based on their ellipticity change, each mutant was grouped into three. Group 1 included mutants S35A, K56A and R39A and exhibited the largest ellipticity change, suggesting the minimum activity change (Figure 3 and Supplementary Figures S4C, E, G and S6). The double or triple mutants belonging to group 2 showed the intermediate displacement, implying that these mutants are functionally less active. Mutants in group 3 appeared to lose their B-to-Z transition activity, which accounts for the residues essential for the B-to-Z transition. For example, the replacement of either Lys34 or Asn38 to alanine in group 3 resulted in dramatic reduction of the B-to-Z transition (Figure 3 and Supplementary Figure S4B and D). In the caZα_PKZ_:Z-DNA complex, P2 and P3 form a wide interaction network with many residues in caZα_PKZ_ (Figure 2C). Asn38, Tyr42, Pro58 and Trp60 are the binding partners of P2, while P3 is recognized by Ser35, Lys34, and Asn38 (Figure 2). Accordingly, mutations of residues in this interaction network severely damage protein:DNA interaction. For example, K34A and N38A showed significantly decreased B-to-Z transition activity (Figure 3). These results suggest P2 and P3 interactions are both crucial for protein:Z-DNA interaction. This plot also proved that Tyr42 and Trp60 have the most critical role in DNA binding since the single mutation at those sites almost completely abolished the B-to-Z transition function (Figure 3 and Supplementary Figure S4F and H). Previously, it was suggested that these mutants stabilize Z-DNA conformation by forming a CH–π interaction (25,26). However, our data suggest that they might be also important for the folding of Zα domains since the alanine mutant of Try42 or Trp60 seems to have the altered secondary structures compared with wild-type protein (Supplementary Figure S5).

**Figure 4.**
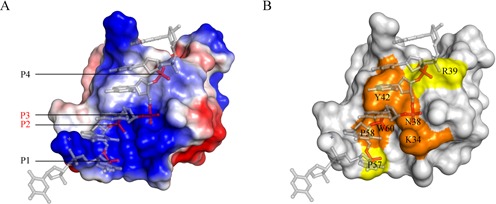
The Z-DNA binding surface of caZα_PKZ_. (**A**) Electrostatic distribution of caZα_PKZ_. Positively charged, negatively charged and hydrophobic regions are depicted in blue, red and white, respectively. P2 and P3 are labeled in red, and P1 and P4 are in black. (**B**) The Z-DNA binding surface of caZα_PKZ_. Key residues involved in P2 and P3 binding are colored orange, and P1 or P4 binding residues are shown in yellow.

Based on the functional study of multisite mutants, we were able to identify the minimal recognition motif of caZα_PKZ_ and interacting phosphates. The K56A/P57A and S35A/K56A/P57A mutants were expected to lose the interaction with P0 and P1, and showed about 50% retention activity relative to the wild-type activity (Figure 3 and Supplementary Figure S7). The R39A/K56A/P57A mutant, which would not interact with P0, P1 and P4, had no detectable B-to-Z transition activity (Figure 3 and Supplementary Figure S7). In addition, the R39A/K56A mutant, in which the interaction with P0 and P4 is abolished, showed about 80% activity (Figure 3 and Supplementary Figure S7). These results indicate that P2 and P3 backbone interactions are not sufficient to stabilize the Z-DNA conformer and suggest that interactions with at least three backbone phosphates, P1, P2 and P3 or P2, P3 and P4, are required. Therefore, the structural geometry of residues involved in either P1, P2 and P3 or P2, P3 and P4 recognition is a likely minimal structural motif of Z-DNA recognition (Figure 4). On the protein side, Lys34, Asn38, Tyr42, Pro58 and Trp60, which are involved in P2 and P3 recognition, Pro57 for P1 binding and Arg39 for P4 binding compose the minimal binding motif for Z-DNA binding (Figure 4).

### B-to-Z transition induced by caZα_PKZ_ is faster than that induced by other Zα domains

**Figure 5.**
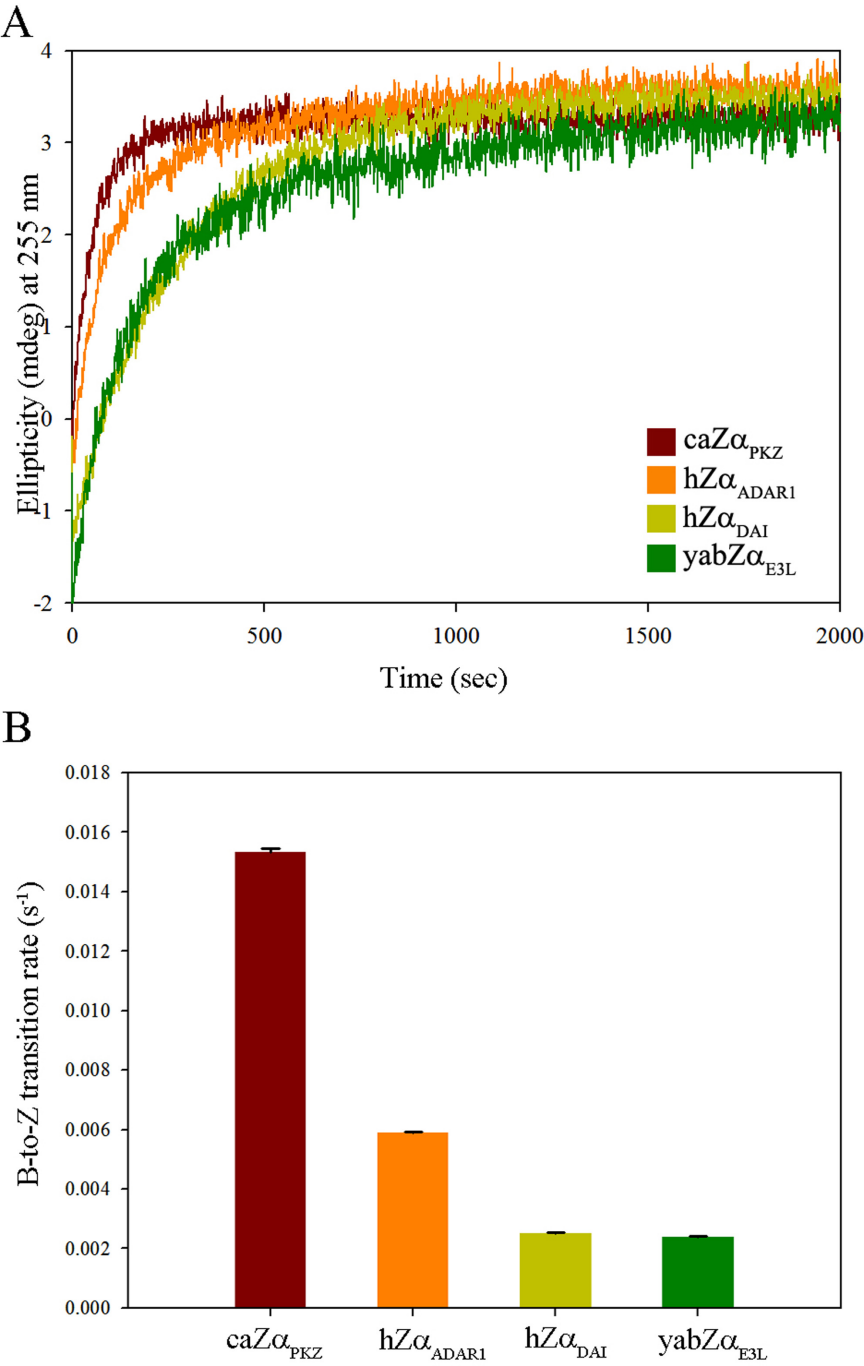
B-to-Z transition kinetics of Zα domains. (**A**) CD spectra of 15 μM of ds-(dCdG)_6_ were monitored at 255 nm in the presence of caZα_PKZ_ (brown), hZα_ADAR1_ (orange), hZα_DAI_ (yellow) and yabZα_E3L_ (green). (**B**) The calculated B-to-Z transition rates (s^−1^) with error bars from caZα_PKZ_ (brown), hZα_ADAR1_ (orange), hZα_DAI_ (yellow) and yabZα_E3L_ (green).

We examined the B-to-Z transition kinetics by measuring the CD spectra at 255 nm for 2000 s. The B-to-Z transition rate and half transition time of ds-(dCdG)_6_ induced by caZα_PKZ_ were calculated to be 15.3 (ms^−1^) and 45.2 s, respectively, by fitting the time course CD using non-linear regression analyses (Figure 5 and Supplementary Table S3). However, B-to-Z transition rates of hZα_ADAR1_, hZα_DAI_ and yabZα_E3L_ were 5.89, 2.51 and 2.39 (ms^−1^), respectively (Figure 5). Accordingly, the half transition times of hZα_ADAR1_, hZα_DAI_ and yabZα_E3L_ were 117.6, 275.9 and 290.5 s, respectively (Supplementary Table S3). Therefore, the B-to-Z transition rate of caZα_PKZ_ is 2.6 times faster than that of hZα_ADAR1_, and 6.1 and 6.4 times faster than those of hZα_DAI_ and yabZα_E3L_, respectively.

### B-to-Z transition activity of caZα_PKZ_ mutants

**Figure 6.**
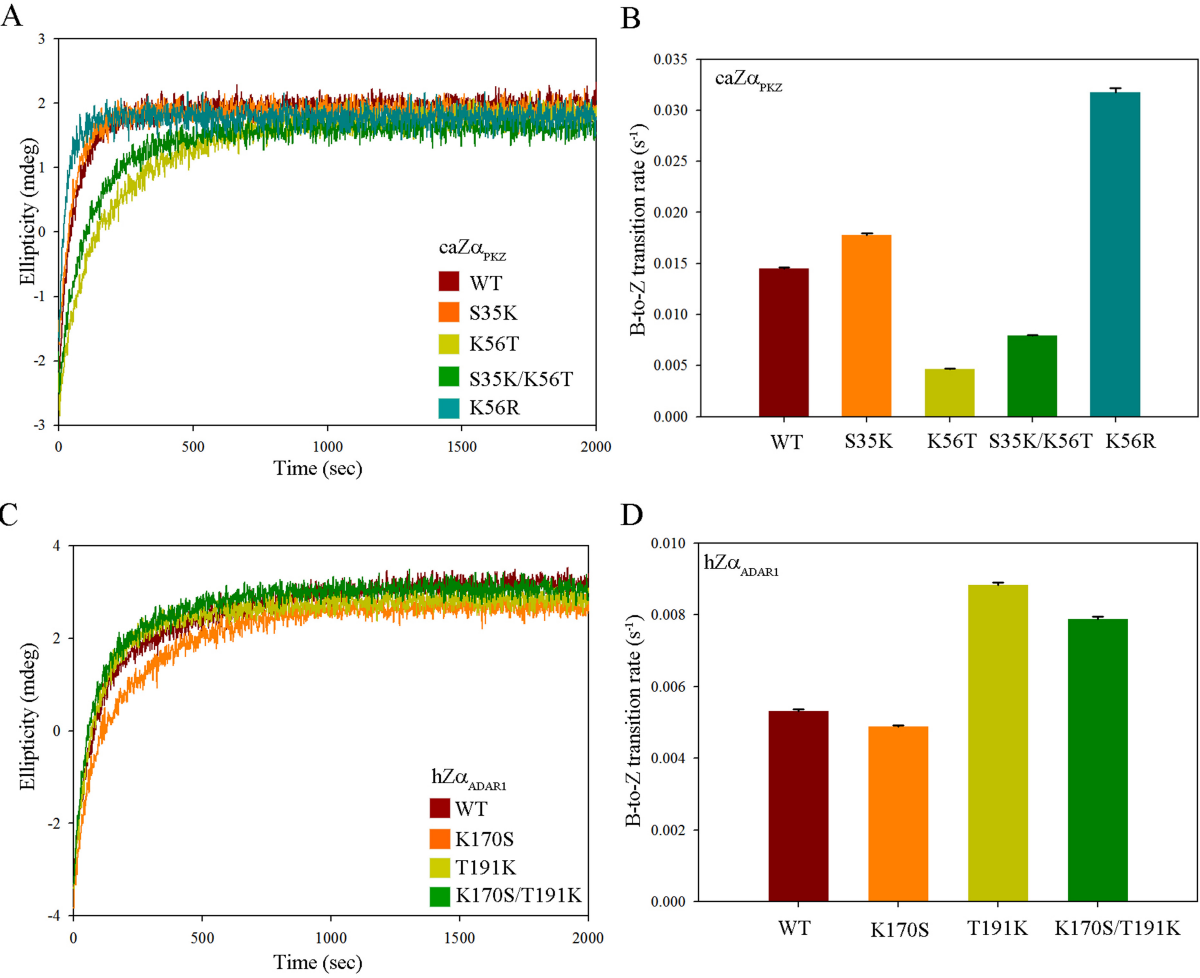
B-to-Z transition activity of caZα_PKZ_ and hZα_ADAR1_. (**A**) Time course CD spectra of caZα_PKZ_ and S35K, K56T, K56R and S35K/K56T mutants. (**B**) The calculated B-to-Z transition rates (s^−1^) of the wild-type, S35K, K56T, S35K/K56T and K56R caZα_PKZ_ are represented by brown, orange, lime, green and turquoise colors, respectively. (**C**) Time course CD spectra of hZα_ADAR1_ and K170S, T191K and K170S/T191K mutants. (**D**) The calculated B-to-Z transition rates (s^−1^) of the wild-type, K170S, T191K and K170S/T191K mutant hZα_ADAR1_ are represented by brown, orange, lime and green colors, respectively.

**Figure 7.**
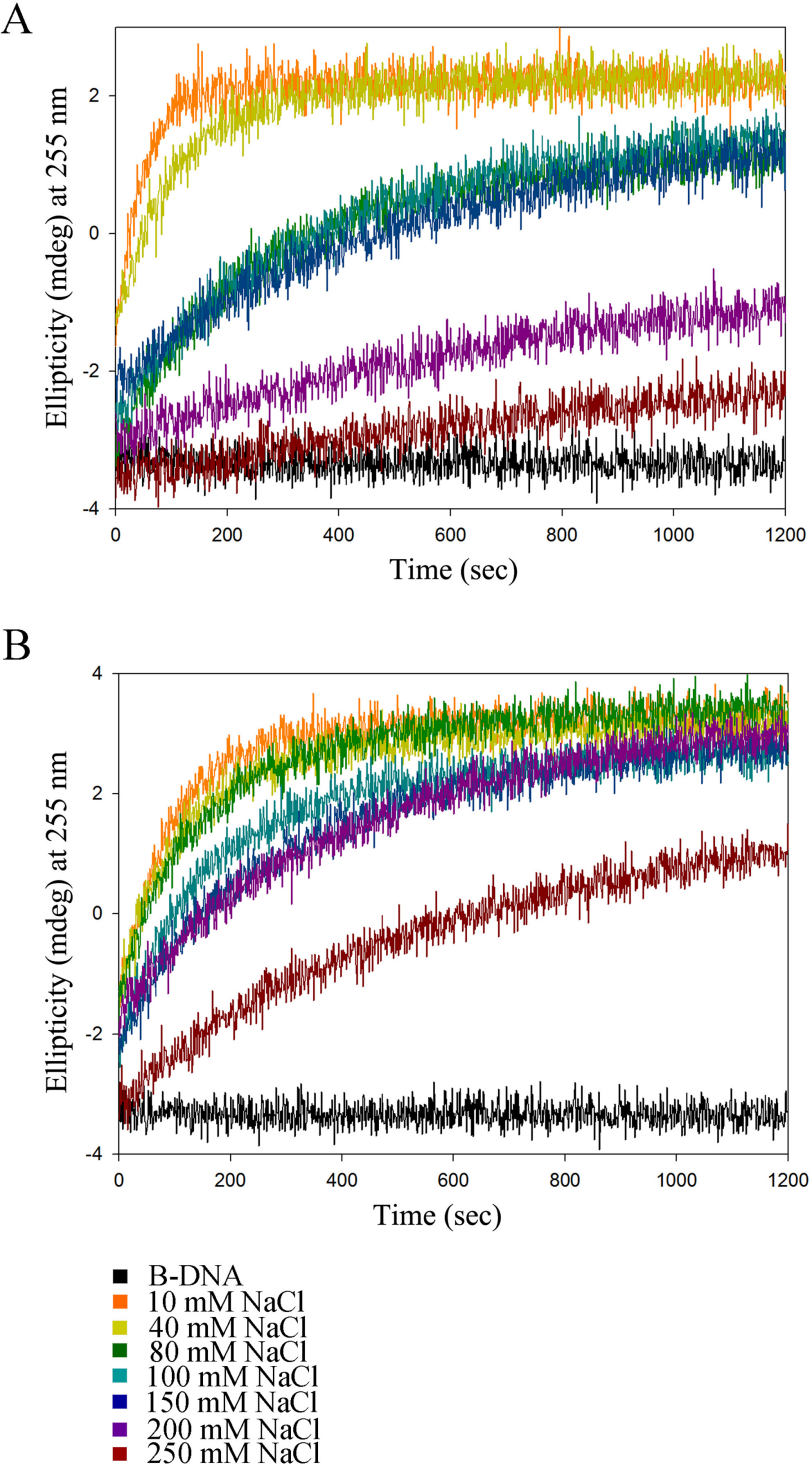
Salt effect on the B-to-Z transition. (**A**) Time course CD spectra of Z-DNA:caZα_PKZ_ in various NaCl concentrations (10, 40, 80, 100, 150, 200 and 250 mM). The ellipticities (mdeg) monitored at 255 nm are indicated by different colors. (**B**) Time course CD spectra of Z-DNA:hZα_ADAR1_ in the same conditions with the same color schemes as in Figure 7A.

The time course B-to-Z transition by caZα_PKZ_ mutants was monitored by CD spectroscopy in order to provide the molecular basis for the high transition rate of caZα_PKZ_. The replacement of Ser35 or Arg39 in the α3 core had only a marginal effect on the B-to-Z transition rate (Supplementary Figure S7 and Supplementary Table S4). However, mutations in residues in the β-wing significantly affected the B-to-Z transition rate; the calculated half transition time was 239 s for the K56A mutant. The activity of the double-mutant K56A/P57A was more drastically reduced, resulting in a half transition time exceeding 527 s (Supplementary Figure S7 and Supplementary Table S4). These results confirmed that the B-to-Z transition rate is largely affected by the interaction between the β-wing of caZα_PKZ_ and Z-DNA. While residues in β-wing affect the B-to-Z transition rate, K34, N38, Y42 and W60 seem to be essential for B-to-Z transition, since their substitution to Ala almost completely demolished the transition (Figure 3 and Supplementary Figures S4 and S7). We further investigated the roles of Ser35 and Lys56 by measuring the transition activity of S35K and K56T mutants since their corresponding residues in hZα_ADAR1_ are Lys and Thr, respectively, in the sequential and structure alignments (Figures 1A and 2). On the other hand, we also made hZα_ADAR1_ mutants by replacing Lys170 and Thr191 with Ser and Lys, respectively, to make caZα_PKZ_-mimicking mutants. The time course CD spectra of caZα_PKZ_ mutants (K56T and S35K/K56T), which have no positive charge at the β-wing, showed a decreased B-to-Z transition rate (Figure 6A and B, and Supplementary Table S5). By contrast, the K56R mutant showed a B-to-Z transition rate that was two times faster than that of wild-type caZα_PKZ_ (Figure 6 and Supplementary Table S3). The caZα_PKZ_-mimicking mutants of hZα_ADAR1_, T191K and K170S/T191K displayed higher B-to-Z transition activity than the wild-type hZα_ADAR1_, while K170S showed the reduced activity (Figure 6C and D, and Supplementary Table S5), although the difference was not as high as that in caZα_PKZ_. These results confirm that charge–charge interaction between the β-wing and DNA backbone is important for the fast kinetics of Zα. Furthermore, we observed that the B-to-Z transition activity was decreased when the reaction was performed in the presence of NaCl (Figure 7), which further demonstrates that the charge–charge interaction is critical for the B-to-Z transition. The reduction of B-to-Z transition rate is more severe in caZα_PKZ_ than in hZα_ADAR1_, suggesting that the effect of charge–charge interaction on B-to-Z transition activity plays a more critical role in the case of caZα_PKZ_ (Figure 7).

### DNA binding kinetics of ZBPs

By functional studies of the mutants, we confirmed that the positive charge in the β-wing can enhance the B-to-Z transition rate. Given this observation, how does the positive charge in the β-wing affect the B-to-Z transition rate? It was proposed that Zα bind to B-DNA prior to the transition to Z-DNA (40,41), so the association rate of Zα to DNA possibly contributes to the B-to-Z transition rate. Under this hypothesis, it is expected that a positively charged residue can enhance the transition rate by increasing the association of negatively charged DNA molecules to the positively charged protein molecules (42). To further analyze the correlation between the association rate and the B-to-Z transition rate, the kinetic parameters for the binding of the caZα_PKZ_, hZα_ADAR1_ and their mutants to DNA were calculated from the Bio-Layer Interferometry measurement of the interaction between DNA and Zα and their correlation with the B-to-Z transition rate (s^−1^) was examined (Supplementary Figures S8, S9 and S10; Supplementary Table S6). We found that B-to-Z transition rates of the wild-type and mutants caZα_PKZ_ are proportional to the association rate constant *k*_on_ (Supplementary Figure S9E) that is also proportional to the number of positively charged residues in the Z-DNA binding surface. A similar correlation is also found in hZα_ADAR1_, although the correlation coefficient (*r*) is relatively lower than that of caZα_PKZ_ (Supplementary Figure S10 for hZα_ADAR1_).

## DISCUSSION

We have compared the Z-DNA binding structure and B-to-Z transition activity of caZα_PKZ_ with those of other Zα domains. A structural comparison revealed not only the conserved overall binding mode of caZα_PKZ_ but also some distinctive features that appear to be important to its higher B-to-Z transition rate. Unlike other ZBDs, caZα_PKZ_ harbors a positively charged residue in the β-wing, Lys56, which plays a key role in DNA binding by forming a wide interaction with DNA, including bonds with both P1 and P0 (Figure 2A and C, and Supplementary Figure S2B).

Although the overall charge distribution is not conserved among ZBDs, positively charged residues and hydrophobic residues are commonly observed in the α3 core and in the β-wing, respectively, which play essential roles in Z-DNA binding. However, it seems that the positively charged residues have a more significant effect on DNA binding and the B-to-Z transition than hydrophobic residues. There are three basic residues involved in Z-DNA recognition in caZα_PKZ_ (Lys34, Arg38 and Lys56) and hZα_ADAR1_ (Lys169, Lys170 and Arg174) (Figure 1A). However, hZα_DAI_ and yabZα_E3L_ have only two positive residues in the Z-DNA binding surface (Figure 1A). Accordingly, hZα_ADAR1_ induces 2.3 and 2.5 times faster B-to-Z transition than hZα_DAI_ and yabZα_E3L_, respectively (Figure 5B). In contrast, the B-to-Z transition rate of caZα_PKZ_, which has one more basic residue in the β-wing than hZα_DAI_ and yabZα_E3L_, is 6.1 and 6.4 times faster than that of hZα_DAI_ and yabZα_E3L_, respectively (Figure 5B and Supplementary Table S3). These results suggest that the contribution of positively charged residues during the B-to-Z transition is more significant when they are present in the β-wing than in the α3 helix. Consistently, the K56T mutation in caZα_PKZ_ significantly lowered the transition rate from 14.5 ms^−1^ to 4.68 ms^−1^ (Figure 6A and B, and Supplementary Table S5), while the addition of a positively charged residue in the β-wing enhanced the activity of hZα_ADAR1_ from 5.32 ms^−1^ to 8.83 ms^−1^ (Figure 6C and D, and Supplementary Table S5). However, the effect of charged residues in the α3 helix seems to be not as significant as the effect of charged residues in the β-wing, although the positive charge in the α3 helix still contributes to an increased B-to-Z transition rate. Furthermore, the S35K mutant showed a higher transition rate than the wild-type caZα_PKZ_ (Figure 6A and B, and Supplementary Table S5), and the K170S mutant showed a slightly decreased B-to-Z transition rate compared to the wild-type hZα_ADAR1_ (Figure 6C and D, and Supplementary Table S5). These results are also consistent with the kinetic study of the viral Zα protein; the positively charged amino acids in the β-wing of Zα_E3L_ enhanced the B-to-Z transition activity (28). Taking these results together, we conclude that the positively charged residue in the β-wing could facilitate the B-to-Z transition (Figure 6). The fastest B-to-Z transition rate of the K56R mutant of caZα_PKZ_ among wild-type and all mutant ZBDs (Figures 5 and 6, and Supplementary Table S3) and the reduction of the transition rate under high salt conditions (Figure 7) also support our conclusion.

From a structural point of view, Arg56 in the model of the K56R mutant of caZα_PKZ_ can fit nicely into the pocket between P0 and P1, suggesting that Arg can form a tight interaction with P0 and P1 groups (Supplementary Figure S11). From the further analyses of their DNA binding kinetics (Supplementary Figures S8–S10), we propose that the increased transition activity is achieved by the enhanced association of ZBDs to DNA through their charge–charge interaction. However, this correlation was not found when different Zα domains were compared (Supplementary Figure S8), which is possibly explained by the fact that the contribution of a positive charge at α3 and the β-wing during the B-to-Z transition is not the same as that shown in Figure 6 and in Supplementary Table S5. Given these results, we conclude that the association rate constant is affected by the presence of a positively charged residue on the DNA binding surface, which eventually enhances the B-to-Z transition rate. Moreover, the contribution of a positive charge in the β-wing is larger than that in the helix α3.

In terms of structure, unlike the other crystal structures of ZBDs complexed with Z-DNA (19,25,31), Mn^2+^ ion is bound to the caZα_PKZ_:Z-DNA complex. It was proposed that nickel and cobalt ions can bind to guanine N7 due to the increased solvent accessibility in the Z-DNA conformation (43,44) and the binding of manganese ion to Z-DNA was recently reported (45). However, among all N7 atoms of guanine in caZα_PKZ_, N7 of G2 appeared to specifically participate in the coordination of the manganese ion. The manganese ion is also coordinated by O1P of C1, which is stabilized by Lys56, so Zα binding is thought to be necessary for coordinating the manganese ion. To investigate the role of manganese ion in the biochemical function of caZα_PKZ_, the B-to-Z transition rate of caZα_PKZ_ was measured in the presence of 15-mM MnCl_2_. However, the manganese ion seems to not contribute to the fast B-to-Z transition kinetics of caZα_PKZ_, since the transition rate decreased from 15.3 (ms^−1^) to 9.49 (ms^−1^). hZα_ADAR1_ and hZα_DAI_ showed marginally increased B-to-Z transition kinetics in the presence of manganese ion (Supplementary Figure S12). Therefore, MnCl_2_ does not seem to be correlated with the fast B-to-Z transition kinetics of caZα_PKZ_, and thus the binding of manganese ion to caZα_PKZ_ may be considered as a crystallization artifact since the crystallization buffer contained 15-mM MnCl_2_.

It has been postulated that the interaction of ZBP to Z-DNA is critical for various biological functions, and thus the B-to-Z transition activity of the ZBPs is relevant to the biological functions. DAI binding to the cytosolic dsDNA turns on the innate immune signal by triggering the activation of NF–kB signaling and expression of type-1 IFN (23). However, PKZ works as an innate immune sensor by recognizing poly-d(GC) as Z-DNA and turning off general translation machinery by phosphorylating eukaryotic translation initiation factor 2α (eIF2α) (8,29). Interestingly, in fish species, both PKZ and PKR are known to play an antiviral role as IFN-inducible eIF2α kinases (46). Therefore, we are intrigued by the functional relationship between PKZ and PKR in terms of the antiviral response. A recent study of PKZ and PKR in *C. auratus* revealed that both proteins work independently, but play a cooperative role in IFN-mediated antiviral response, although antiviral ability of fish PKZ was weaker than that of fish PKR (21). However, the pull-down experiment with poly I:C, a dsRNA mimic, showed that the DNA binding domain of PKZ does not bind to poly I:C, while PKR does bind (21). These results suggest that PKR and PKZ might independently contribute to the innate immune response by recognizing different nucleotide substrates in the initial stage of the innate immune response. Indeed, PKZ-dependent phosphorylation of eIF2α and the subsequent translational shutdown are largely induced not by dsRNA but by Z-DNA (8,29). Since the Z-DNA-induced innate immune responses conducted by DAI could be regulated by competition with other ZBPs such as ADAR1 or E3L, differential Z-DNA binding activity or the B-to-Z transition rate of Zα domains in the innate immune sensors might be a key to controlling the innate immune response. Similarly, since fish virus also harbor the ZBPs (47), it is also expected that PKZ must compete with viral ZBPs to play a role in fish innate immune response during viral infection. Therefore, the fast Z-DNA converting activity of PKZ might be necessary for competing with viral ZBPs. Further studies on the B-to-Z transition activity of Zα domains in cellular environments will clearly define the biological relevance of the structural and kinetic differences between caZα_PKZ_ and other Zα domains. In addition, structural and functional studies on intact ZBPs are also required for better understanding of the Z-DNA-dependent innate immune response.

The current study also provides an insight into a novel ZBD. Z-DNA is known to have various roles in cellular events. Z-DNA formation is necessary for opening chromatin structure and activating transcription of target genes (48,49). Z-DNA is also known to cause genomic instability (50). For the regulation of diverse functions of Z-DNA, more ZBPs with novel ZBDs are expected to mediate various cellular processes associated with Z-DNA. The results from this study combined with those from structural and functional studies of Zα_ADAR1_ confirm that Z-DNA can be recognized by Zα proteins through three backbone phosphates, but the interaction modes for caZα_PKZ_ and Zα_ADAR1_ are not the same in detail (39). Considering the size and charge distribution of the minimum unit of Z-DNA for protein binding and the diversity of the Z-DNA binding mode, it is expected that more ZBPs other than the Zα domain may be present in cells. Consistently, this possibility is partially proven by identifying putative ZBPs that interact with the Z-form of ADAM-12 NRE sequence (51). Therefore, the current study not only reveals a structural requirement for fast B-to-Z transition but also provides insight into the presence of novel ZBPs.

## ACCESSION NUMBERS

PDB IDs: 4KMF, 1QBJ, 1J75, 1SFU, 3EYI.

## SUPPLEMENTARY DATA

Supplementary Data are available at NAR Online, including [1–5].

## FUNDING

Samsung Science and Technology Foundation [SSTF-BA1301-01 to K.K.K.]; National Research Foundation [2009-0075300 to Y.G.K.]. Funding for open access charge: Samsung Science and Technology Foundation [SSTF-BA1301-01]; NRF [2009-0075300].

*Conflict of interest statement*. None declared.

## Supplementary Material

SUPPLEMENTARY DATA
