# Supplementary material for: Distinct Z-DNA binding mode of a PKR-like protein kinase containing a Z-DNA binding domain (PKZ)
Source: Nucleic Acids Res. 2014 Mar 20;42(9):5937–48. doi: 10.1093/nar/gku189 (PMC4027156; doi:10.1093/nar/gku189)
Supplement: SUPPLEMENTARY DATA [file supp_42_9_5937__index.html]

Distinct Z-DNA binding mode of a PKR-like protein kinase containing a Z-DNA binding domain (PKZ) — SUPPLEMENTARY DATA 

# Distinct Z-DNA binding mode of a PKR-like protein kinase containing a Z-DNA binding domain (PKZ)

## SUPPLEMENTARY DATA

**Files in this Data Supplement:**

- SUPPLEMENTARY DATA
