# Supplementary material for: Distinct Z-DNA binding mode of a PKR-like protein kinase containing a Z-DNA binding domain (PKZ)
Source: Nucleic Acids Res. 2014 Mar 20;42(9):5937–48. doi: 10.1093/nar/gku189 (PMC4027156; doi:10.1093/nar/gku189)

**The distinct Z-DNA binding mode of a PKR-like protein kinase containing Z-DNA binding domain (PKZ)**

Doyoun Kim^1^, Jeonghwan Hur^1^, Kwangsoo Park^1^, Sangsu Bae^2,3^, Donghyuk Shin^4^, Sung Chul Ha^5^, Hye-Yeon Hwang^1^, Sungchul Hohng^2,3,6^, Joon-Hwa Lee^7^, Sangho Lee^4^, Yang-Gyun Kim^8*^, and Kyeong Kyu Kim^1*^

^1^Department of Molecular Cell Biology, Samsung Biomedical Research Institute, Sungkyunkwan University School of Medicine, Suwon 440-746, Korea, ^2^Department of physics and Astronomy, ^3^National Center for Creative Research Initiatives, ^4^Department of Biological Sciences, Sungkyunkwan University, Suwon 440-746, Korea, ^5^Pohang Accelerator Laboratory, Pohang University of Science and Technology, Pohang, Kyungbuk 790-784, Korea, ^6^Department of Biophysics and Chemical Biology Seoul National University, Seoul 151-747, Korea, ^7^Department of Chemistry and RINS, Gyeongsang National University, Jinju, Gyeongnam 660-701, Republic of Korea, and ^8^Department of Chemistry, Sungkyunkwan University, Suwon 440-746, Korea

^*^To whom correspondence should be addressed. E-mail: [kyeongkyu@skku.edu](mailto:kyeongkyu@skku.edu) (K. K. Kim); [ygkimmit@skku.edu](mailto:ygkimmit@skku.edu) (Y.G. Kim)

**Contents:**

Supplementary Materials and Methods

Supplementary References

Supplementary Table S1-S6

Supplementary Figure Legends

Supplementary Figure S1-S12

**Supplementary Materials and Methods**

**Protein preparation** The coding sequence for residue 1-75 of caZα_PKZ_ was cloned into an *E. coli* expression plasmid pET28a (Novagen, WI, USA). PCR for site-directed mutagenesis was performed using designed primers and caZα_PKZ_-pET28a as a template. *E.coli* BL21(DE3) (Novagen, WI, USA) transformed with the recombinant plasmid was grown in Luria-Bertani media containing 30 μg ml^-1^ kanamycin at 37℃ and 1 mM isopropyl-β-D-thiogalactoside (IPTG) was added when the OD_600_ reached 0.6. Cells were harvested after four hours and caZα_PKZ_ was purified as described elsewhere ([1](#_ENREF_31)). Briefly, after the initial chromatography on a HiTrap metal-chelating column (GE Healthcare, NJ, USA), and thrombin treatment, caZα_PKZ_ was further purified using a ResourceS ion-exchange column (GE Healthcare, NJ, USA). The purified protein was dialyzed against buffer A (5 mM HEPES pH 7.5 and 10 mM NaCl) and concentrated to 1 mM. The Zα domain from human DAI (hZα_DAI_) was purified in the same way (2). The Zα domain from human ADAR1 (hZα_ADAR1_), and the Zα domain from the E3L homolog of the yaba-like disease virus (yabZα_E3L_) were also prepared as reported previously (3,4). The concentrations of purified proteins were measured spectroscopically at 280 nm using the extinction coefficients of 6990 M^-1^cm^-1^ for caZα_PKZ_, hZα_ADAR1_ and hZα_DAI_, or 10095 M^-1^cm^-1^ for yabZα_E3L_.

**Calculation of B-to-Z transition rate, half transition rate constant, and time constant** We assumed that the B-to-Z transition by Zα domains is a first-order reaction, since the Z-DNA formation increased in a curvilinear manner with time. Thus, we used non-linear regression analysis to calculate the B-to-Z transition rate constant (s^-1^) from the time-course CD spectra of the B-to-Z transition. The non-linear regression analysis was carried out using the Y = Y_0_+(Y_max_-Y_0_) X (1-e^-kx^) equation, where Y_max_ and Y_0_ are Y values at infinite and zero time, respectively, and k is the B-to-Z transition rate constant (s^-1^). The half transition time, a time scale by which the half B-form DNA is converted to Z-form, was calculated by ln2/k, and the time constant was calculated by 1/k.

**Biolayer interferometry (BLI)** The biolayer interferometry (BLI) experiments were performed using the BLITZ system (Fortebio, CA, USA). Oligonucleotides of 5’- biotin–dAdTdTdAdTdAdT (dCdG)_10_-3’ and 5’-(dCdG)_10_-3’ were purchased (IDT, CA, USA) and annealed in buffer A. The annealed DNA was immobilized into the streptavidin-coated biosensor (Fortebio, CA, USA). The DNA immobilized biosensors were equilibrated with buffer A, and reacted with various concentrations of caZα_PKZ_ and mutants(2.5 μM to 10 μM). Similarly, the binding of other Zα proteins and mutants to DNA was tested with the DNA-immobilized biosensors. The equilibrium binding constant (*K*_d_), association rate constant (*k*_on_), and dissociation rate constant (*k*_off_) were determined from the BLI data at various concentrations using the global fitting method provided in data analysis software version 7.0 (Fortebio, CA, USA).

**Supplementary References**

1. Kim, D., Hwang, H.Y., Kim, Y.G. and Kim, K.K. (2009) Crystallization and preliminary X-ray crystallographic studies of the Z-DNA-binding domain of a PKR-like kinase (PKZ) in complex with Z-DNA. *Acta Crystallogr Sect F Struct Biol Cryst Commun*, **65**, 267-270.

2. Schwartz, T., Behlke, J., Lowenhaupt, K., Heinemann, U. and Rich, A. (2001) Structure of the DLM-1-Z-DNA complex reveals a conserved family of Z-DNA-binding proteins. *Nat Struct Biol*, **8**, 761-765.

3. Schwartz, T., Rould, M.A., Lowenhaupt, K., Herbert, A. and Rich, A. (1999) Crystal structure of the Zalpha domain of the human editing enzyme ADAR1 bound to left-handed Z-DNA. *Science*, **284**, 1841-1845.

4. Ha, S.C., Lokanath, N.K., Van Quyen, D., Wu, C.A., Lowenhaupt, K., Rich, A., Kim, Y.G. and Kim, K.K. (2004) A poxvirus protein forms a complex with left-handed Z-DNA: crystal structure of a Yatapoxvirus Zalpha bound to DNA. *Proceedings of the National Academy of Sciences of the United States of America*, **101**, 14367-14372.

5. Lu, X.J. and Olson, W.K. (2003) 3DNA: a software package for the analysis, rebuilding and visualization of three-dimensional nucleic acid structures. *Nucleic acids research*, **31**, 5108-5121.

**Supplementary Tables**

**Supplementary Table S1. Base pair step parameters of Z-DNA in complex with caZα_PKZ_, hZα_ADAR1_ and B-DNA.**

| **Base step** | **Shift** | **Slide** | **Rise** | **Tilt** | **Roll** | **Twist** |
| --- | --- | --- | --- | --- | --- | --- |
| C1-G2 | 0.04 | 5.35 | 3.51 | -1.82 | -4.46 | -4.38 |
| G2-C3 | 0.01 | -1.45 | 3.17 | -0.36 | -8.11 | -45.83 |
| C3-G4 | 0.00 | 5.33 | 3.73 | -0.00 | -6.99 | -8.63 |
| G4-C5 | -0.01 | -1.45 | 3.17 | 0.36 | -8.11 | -45.83 |
| C5-G6 | -0.04 | 5.35 | 3.51 | 1.82 | -4.46 | -4.38 |
| **Averaged** |  |  |  |  |  |  |
| caZα_PKZ_ | 0.00 | 2.63 | 3.42 | 0.00 | -6.43 | -21.81 |
| hZα_ADAR1_ | -0.05 | 2.79 | 3.52 | -0.38 | -3.80 | -25.31 |
| B-DNA | -0.02 | 0.12 | 3.36 | -0.19 | 0.02 | 35.58 |

Each parameter was calculated using software 3DNA (5). For standard comparison, the parameters of B-DNA and Z-DNA from hZα_ADAR1_ were calculated from crystal structure of B-DNA (PDB ID : 1BNA) and Z-DNA : hZα_ADAR1_ (PDB ID : 1QBJ) complex.

**Supplementary Table S2. Sugar conformations of Z-DNA in complex with caZα_PKZ_ and hZα_ADAR1_.**

| **Z-DNA from caZα_PKZ_** | | **Z-DNA from hZα_ADAR1_** | |
| --- | --- | --- | --- |
| **Base** | **Puckering** | **Base** | **Puckering** |
| C1 | C2’ - endo | C1 | C2’ - endo |
| G2 | C4’ - exo | G2 | C3’ - endo |
| C3 | C2’ - endo | C3 | C2’ - endo |
| G4 | C3’ - endo | G4 | C3’ - endo |
| C5 | C2’ - endo | C5 | C2’ - endo |
| G6 | C4’ –exo | G6 | C3’ - endo |

Each parameter was calculated using software 3DNA ([1](#_ENREF_1)). Z-DNA form crystal structure of Z-DNA : hZα_ADAR1_ (PDB ID : 1QBJ) was used for comparison study.

**Supplementary Table S3. The kinetic parameters of caZα_PKZ_, hZα_ADAR1_, hZα_DAI_, and yabZα_E3L_.**

|  | B-to-Z Transition  rate constant, k (s^-1^) | Half transition time (sec) | Time constant,  (sec) |
| --- | --- | --- | --- |
| caZα_PKZ_ | 1.53e-02 ± 1.28e-04 | 45.19 | 65.21 |
| hZα_ADAR1_ | 5.89e-03 ± 3.94e-05 | 117.59 | 169.69 |
| hZα_DAI_ | 2.51e-03 ± 1.67e-05 | 275.99 | 398.26 |
| yabZα_E3L_ | 2.39e-03  ± 2.09e-05 | 290.47 | 419.14 |
| caZα_PKZ_ K56R | 3.18e-02 ± 4.26e-04 | 21.82 | 31.48 |

**Supplementary Table S4. The kinetic parameters of caZα_PKZ_ and its mutants, R39A, S35A, K56A, K56A/P57A, S35A/K56A/P57A, R39A/K56A, and S35A/R39A/K56A.**

|  | B-to-Z Transition  rate constant, k (s^-1^) | Half transition time (sec) | Time constant,  (sec) |
| --- | --- | --- | --- |
| WT | 1.53e-02 ± 1.28e-04 | 45.19 | 65.21 |
| R39A | 9.49e-03 ± 5.40e-05 | 73.05 | 105.37 |
| S35A | 8.45e-03 ± 4.16e-05 | 82.06 | 118.34 |
| K56A | 2.90e-03 ± 1.81e-05 | 239.06 | 344.82 |
| K56A/P57A | 1.32e-03 ± 7.36e-06 | 527.01 | 757.57 |
| S35A/K56A/P57A | 1.13e-03 ± 6.91e-06 | 612.59 | 884.95 |
| R39A/K56A | 1.00e-03 ± 4.93e-06 | 691.62 | 1000.00 |
| S35A/R39A/K56A | 9.04e-04 ± 6.63e-06 | 766.93 | 1106.19 |

**Supplementary Table S5. The kinetic parameters of caZα_PKZ_, hZα_ADAR1_, and its mimicking mutants.**

|  | B-to-Z Transition  rate constant, k (s^-1^) | Half transition time (sec) | Time constant,  (sec) |
| --- | --- | --- | --- |
| caZα_PKZ_ |  |  |  |
| WT | 1.45e-02 ± 1.27e-04 | 47.84 | 69.03 |
| S35K | 1.78e-02 ± 1.82e-04 | 38.99 | 56.26 |
| K56T | 4.68e-03 ± 2.89e-05 | 148.14 | 213.76 |
| S35K/K56T | 7.93e-03 ± 5.62e-05 | 87.36 | 126.06 |
| hZα_ADAR1_ |  |  |  |
| WT | 5.32e-03 ± 5.68e-05 | 130.36 | 188.11 |
| K170S | 4.88e-03 ± 3.78e-05 | 142.142 | 205.11 |
| T191K | 8.83e-03 ± 7.19e-05 | 78.53 | 113.31 |
| K170S/T191K | 7.88e-03 ± 7.39e-05 | 87.89 | 126.83 |

**Supplementary Table S6. Parameters of the Bio-Layer Interferometry experiment from Zα domains and mimic mutants.**

|  | *K*_d_ (M) | *k*_on_ (1/Ms) | *k*_off_ (1/s) |
| --- | --- | --- | --- |
| caZα_PKZ_ WT | 8.51e-07 | 5.28e+04 ± 9.74e+02 | 4.49e-02 ± 6.70e-04 |
| caZα_PKZ_ S35K | 2.98e-07 | 8.72e+04 ± 2.46e+03 | 2.60e-02 ± 7.49e-04 |
| caZα_PKZ_ K56T | 6.58e-07 | 1.60e+04 ± 3.84e+02 | 1.05e-02 ± 2.15e-04 |
| caZα_PKZ_ S35K/K56T | 1.23e-06 | 4.00e+04 ± 9.34e+02 | 4.93e-02 ± 9.36e-04 |
| hZα_ADAR1_WT | 1.86e-07 | 6.66e+04 ± 1.11e+03 | 1.24e-02 ± 2.92e-04 |
| hZα_ADAR1_ K170S | 5.24e-07 | 6.87e+04 ± 1.38e+03 | 3.60e-02 ± 5.73e-04 |
| hZα_ADAR1_ T191K | 3.71e-07 | 7.51e+04 ± 1.47e+03 | 2.79e-02 ± 5.02e-04 |
| hZα_ADAR1_ K170S/T191K | 2.94e-07 | 8.80e+04 ± 1.91e+03 | 2.59e-02 ± 4.82e-04 |
| hZα_DAI_ | 4.94e-07 | 5.17e+04 ± 9.00e+02 | 2.55e-02 ± 3.43e-04 |
| yabZα_E3L_ | 8.72e-08 | 1.09e+04 ± 2.50e+02 | 9.47e-04 ± 5.72e-05 |

**Supplementary Figure Legends**

**Supplementary Figure S1. Structural comparison of Zα domains**. A. Root Mean Square Deviation (RMSD) vs. residues plot. RMSDs between caZα_PKZ_ and hZα_ADAR1_ (PDB ID : 1QBJ), mZα_DAI_ (PDB ID : 1J75), yabZα_E3L_ (PDB ID : 1SFU), and hZβ_DAI_ (PDB ID : 3EYI) are colored brown, orange, yellow, and green, respectively. Unaligned residues are marked with “-1”. Structural superposition of caZα_PKZ_ with hZα_ADAR1_ (B), mZα_DAI_ (C), yabZα_E3L_ (D), and hZβ_DAI_ (E) with ribbon models. The compared structures were colored by the RMSD scale from blue (low RMSD) to red (high RMSD). The color of the RMSD scale is shown in the right panel with RMSD in Å.

**Supplementary Figure S2. Electron density maps of Z-DNA.** **A.** Single stranded DNA is shown by ball and stick models with the *2Fo-Fc* electron density map contoured at 1.5 . DNA backbones and bases are colored red and gray, respectively. All DNA bases are labeled. **B.** Electron density map of the manganese ion with the coordinated atoms. Manganese ion is octahedrally coordinated by N7 of G2, O1P of C1, and four water molecules. A manganese atom and water molecules are represented by magenta and green spheres, respectively. *2Fo-Fc* electron density map for the manganese ion and water molecules are contoured at 9.0  and 2.0 , respectively. The N7 of guanine 2, O1P of cytosine 1, manganese ion, and four water molecules are labeled as N7, O1P, Mn, and W, respectively. The distances between manganese ion and the coordinated atoms are indicated in an angstrom unit.

**Supplementary Figure S3. The electrostatic charge distribution of caZα_PKZ_ (A), hZα_ADAR1_ (B), mZα_DAI_ (C), and yabZα_E3L_ (D)**. Wing structures of Zα domains are indicated by a dark triangle. Blue and red colors represent positively and negatively charged surfaces scaled from −50 to +50 kT e^−1^. The electrostatic surfaces of each protein are calculated by PyMol software.

**Supplementary Figure S4. B-to-Z transition by caZα_PKZ_ and its mutants.** CD spectra of 7.5 μM of ds(dCdG)_6_ in the presence of various amounts of the wild type caZα_PKZ_ (A) and its mutants, K34A (B), S35A (C), N38A (D), R39A (E), Y42A (F), K56A (G), W60A (H), R39A/K56A (I), R39A/K56A/P57A (J), S35A/K56A/P57A (K), S35A/R39A/K56A (L), S35A/R39A/K56A/P57A (M), and K56A/P57A (N) are monitored The ratio between protein and DNA ([P]/[N] ratio) are 0, 2, 4, 6, and 8.

**Supplementary Figure S5. CD spectra of caZα_PKZ_ mutants** (190-240 nm)**.** (A) CD spectra of the wild-type and mutant caZα_PKZ_ are drawn as solid lines with different colors and labels. The mutants used in this figures are K34A, S35A, N38A, R39A, K56A, R39A/K56A, S35A/R39A/K56A, K56A/P57A, S35A/K56A/P57A, R39A/K56A/P57A, and S35A/R39A/K56A/P57A. **(B)** CD spectra of Y42A and W60A mutants of caZα_PKZ_ are compared with that of the wild type caZα_PKZ._

**Supplementary Figure S6. B-to-Z transition induced by caZα_PKZ_ mutants at various [P]/[N] ratios.** Circular dichroism values at 255 nm **(A)** and 292 nm **(B)** of wild-type caZα_PKZ_ and mutant caZα_PKZ_ in Group1 (S35A, R39A, and K56A) measured at various [P]/[N] ratios are plotted. (C-D) The circular dichroism spectra of wild-type caZα_PKZ_ and mutant caZα_PKZ_ in Group2 (K56A/P57A, R39A/K56A, and S35A/K56A/P57A) measured at various [P]/[N] ratios are plotted. (E-F) The circular dichroism spectra of wild-type caZα_PKZ_ and mutant caZα_PKZ_ in Group 3 (K34A, N38A, Y42A, W60A, S35A/R39A/K56A, R39A/K56A/P57A, and S35A/R39A/K56A/P57A) measured at various [P]/[N] ratios are plotted. CD values were measured 1 hour after mixing protein and DNA. Spectra at 255 nm and 292 nm represent Z-DNA and B-DNA, respectively. Each value is the average of three replicates. Standard deviations are depicted as error bars.

**Supplementary Figure S7. B-to-Z transition kinetics of caZα_PKZ_ and its mutants**. The CD spectra at 255 nm were monitored for an hour after mixing 15 μM of ds(dCdG)_6_ with 60 μM of caZα_PKZ_ and its mutants, K34A, S35A, N38A, R39A, K56A, R39A/K56A, S35A/R39A/K56A, K56A/P57A, S35A/K56A/P57A, R39A/K56A/P57A, and S35A/R39A/K56A/P57A. Each spectrum is indicated with different colors in a box.

**Supplementary Figure S8. The DNA Binding kinetics of Zα domains. **Bio-Layer Interferometry (BLI) sensograms of DNA binding to caZα_PKZ_ **(A)**, hZα_ADAR1_ **(B),** hZα_DAI_ **(C),** and yabZα_E3L_ **(D)** at various concentrations.The solid and broken lines stand for BLI signals from experiments and fitted regression, respectively. The protein concentration of each experiment is indicated by a different color. The DNA binding kinetic parameters, k_on_ **(E),** k_off_ **(F),** and K_d_ **(G)** of caZα_PKZ_, hZα_ADAR1_, hZα_DAI_, and yabZα_E3L_ are plotted with their B-to-Z transition rate (s^-1^). The correlation coefficient is represented as ‘r’.

**Supplementary Figure S9. The DNA Binding kinetics of caZα_PKZ_ and mutatns. **BLI sensograms of DNA binding to caZα_PKZ_ **(A),** and its mutants S35K **(B),** K56T **(C),** and S35K/K56T **(D)** at various concentrations. The solid and broken lines stand for BLI signals from experiments and fitted regression, respectively. The protein concentration of each experiment is indicated by a different color. The DNA binding kinetic parameters, k_on_ **(E),** k_off_ **(F),** and K_d_ **(G)** of caZα_PKZ_ and mutants (S35K, K56T, and S35K/K56T) are plotted with their B-to-Z transition rate (s^-1^). The correlation coefficient is represented as ‘r’.

**Supplementary Figure S10. The DNA Binding kinetics of hZα_ADAR1_ and mutatns. **BLI sensograms of DNA binding to the wild-type hZα_ADAR1_ **(A),** and its mutants K170S **(B),** T191K **(C),** and K170S/T191K **(D)** at various concentrations. The solid and broken lines stand for BLI signals from experiments and fitted regression, respectively. The protein concentration of each experiment is indicated by a different color. The DNA binding kinetic parameters, k_on_ **(E),** k_off_ **(F),** and K_d_ **(G)** of hZα_ADAR1_, and its mutants (K170S, T191K, and K170S/T191K) are plotted with their B-to-Z transition rate (s^-1^). The correlation coefficient is represented as ‘r’.

**Supplementary Figure S11. The model of the K56R mutant of caZα_PKZ_ in complex with DNA.** The model was built by replacing Lys with Arg in the crystal structure of caZα_PKZ_:Z-DNA complex and minimizing the energy. The DNA binding interface of protein is depicted as a blue ribbon and ball-and-stick models. The substituted Arg (Arg56) and contacting phosphate groups (P0 and P1) are labeled. Putative interactions between Arg56 and phosphate groups are depicted as dotted lines. The backbone and bases of DNA are drawn as red and gray ball-and-stick models, respectively. The manganese ion is represented by a magenta sphere.

**Supplementary Figure S12. The effect of manganese ion on the B-to-Z transition kinetics of caZα_PKZ_, hZα_ADAR1_, and hZα_DAI_. A.** Time course CD spectra of caZα_PKZ_, hZα_ADAR1_, and hZα_DAI_ at 255 nm with and without MnCl_2_. **B.** The B-to-Z transition rates (s^-1^) of caZα_PKZ_, hZα_ADAR1_, and hZα_DAI_ with and without MnCl_2_. The black and gray bars stand for the transition rates in the absence and presence of MnCl_2_, respectively.

**Supplementary Figure S1.**


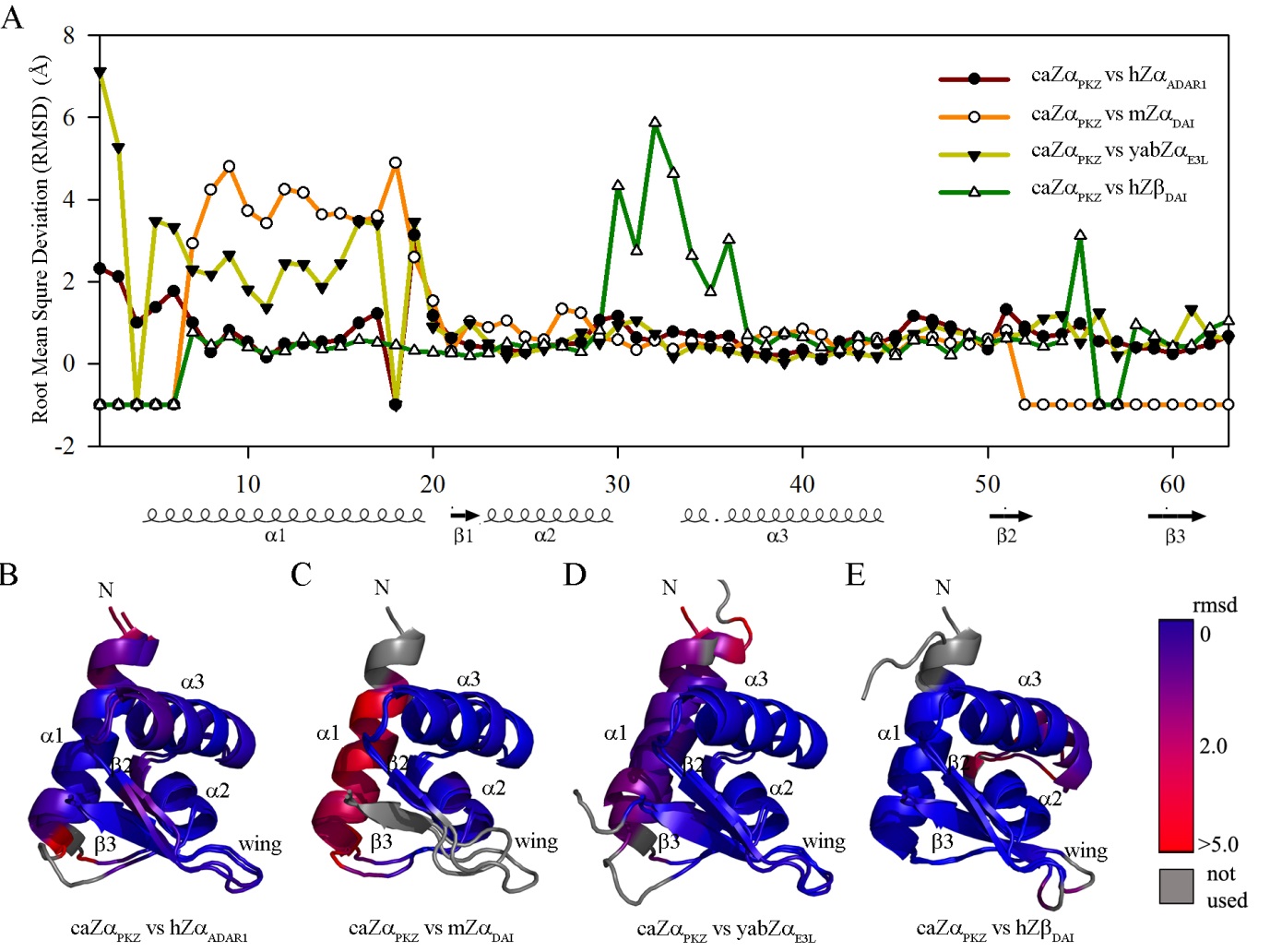


**Supplementary Figure S2**


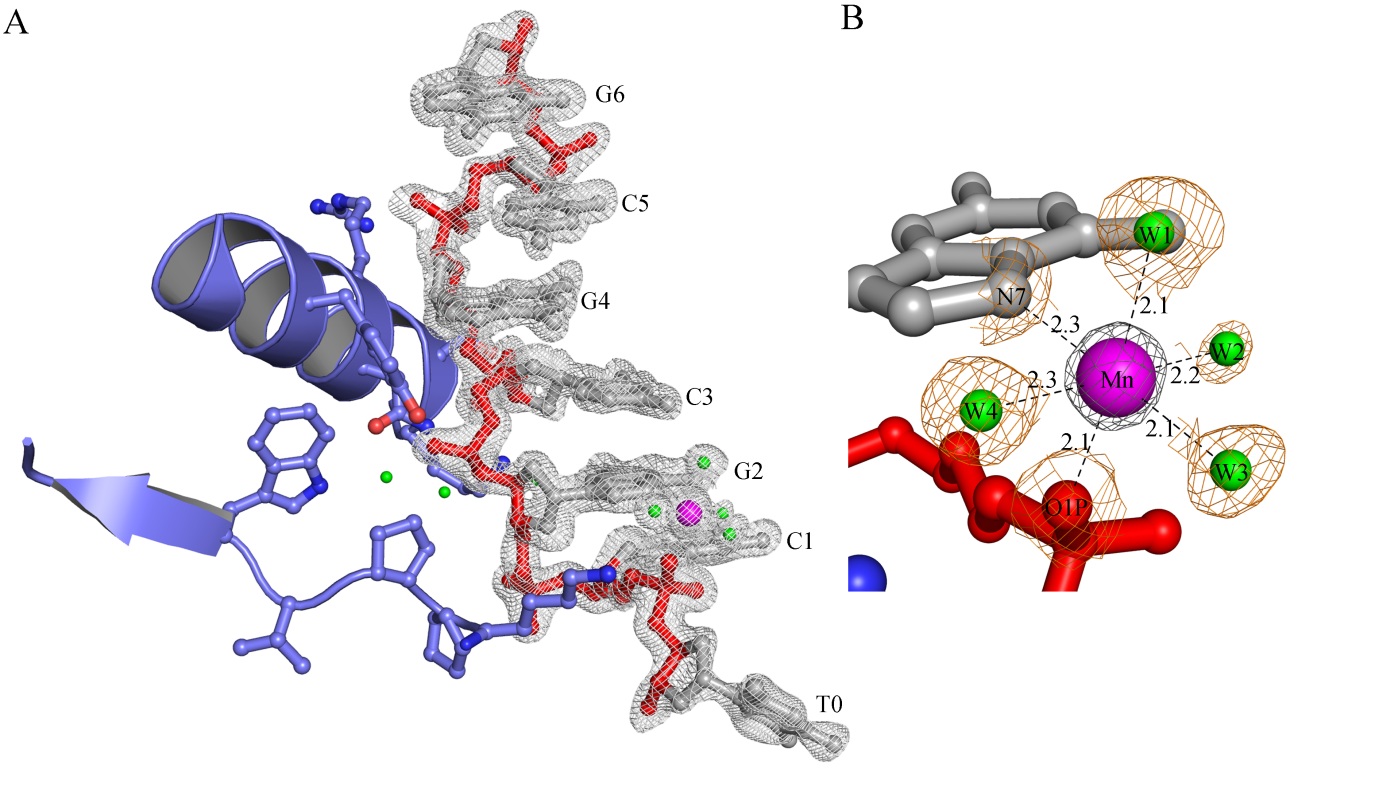


**Supplementary Figure S3**


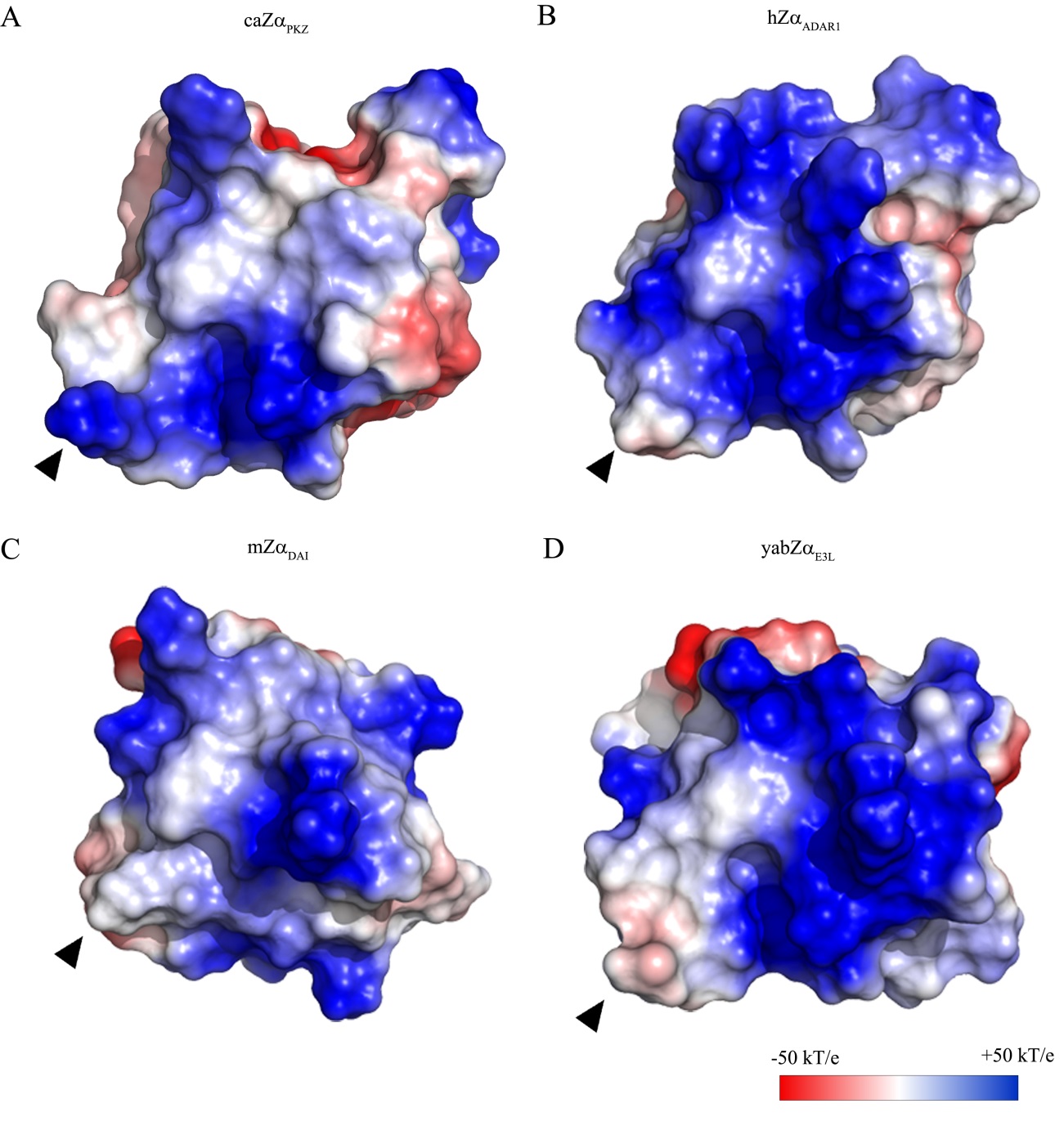


**Supplementary Figure S3.**

**Supplementary Figure S4**


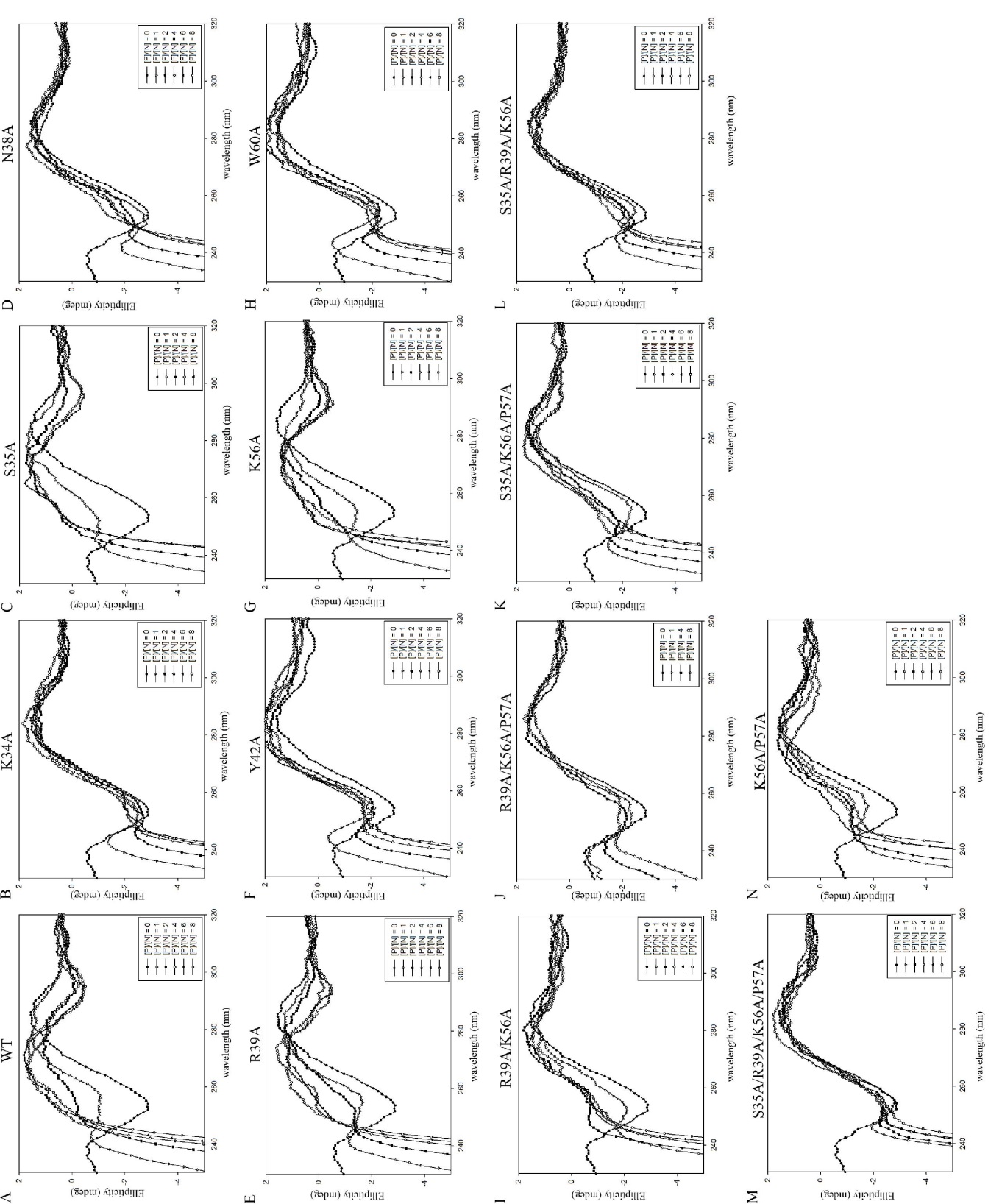


**Supplementary Figure S5**

**
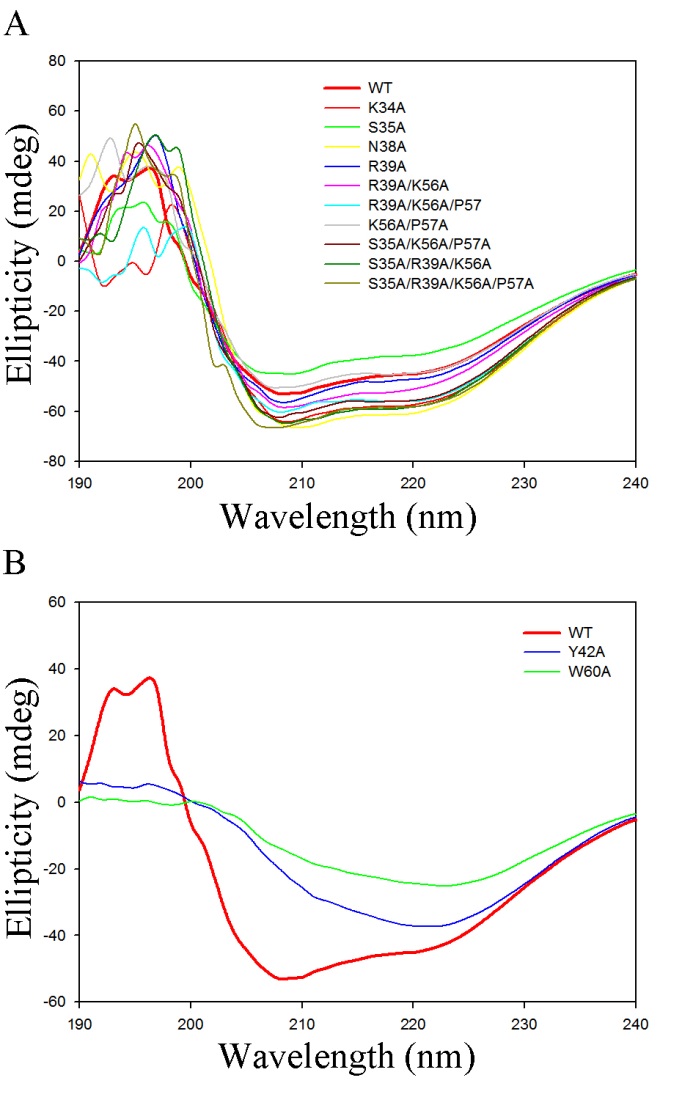
**

**Supplementary Figure S6**


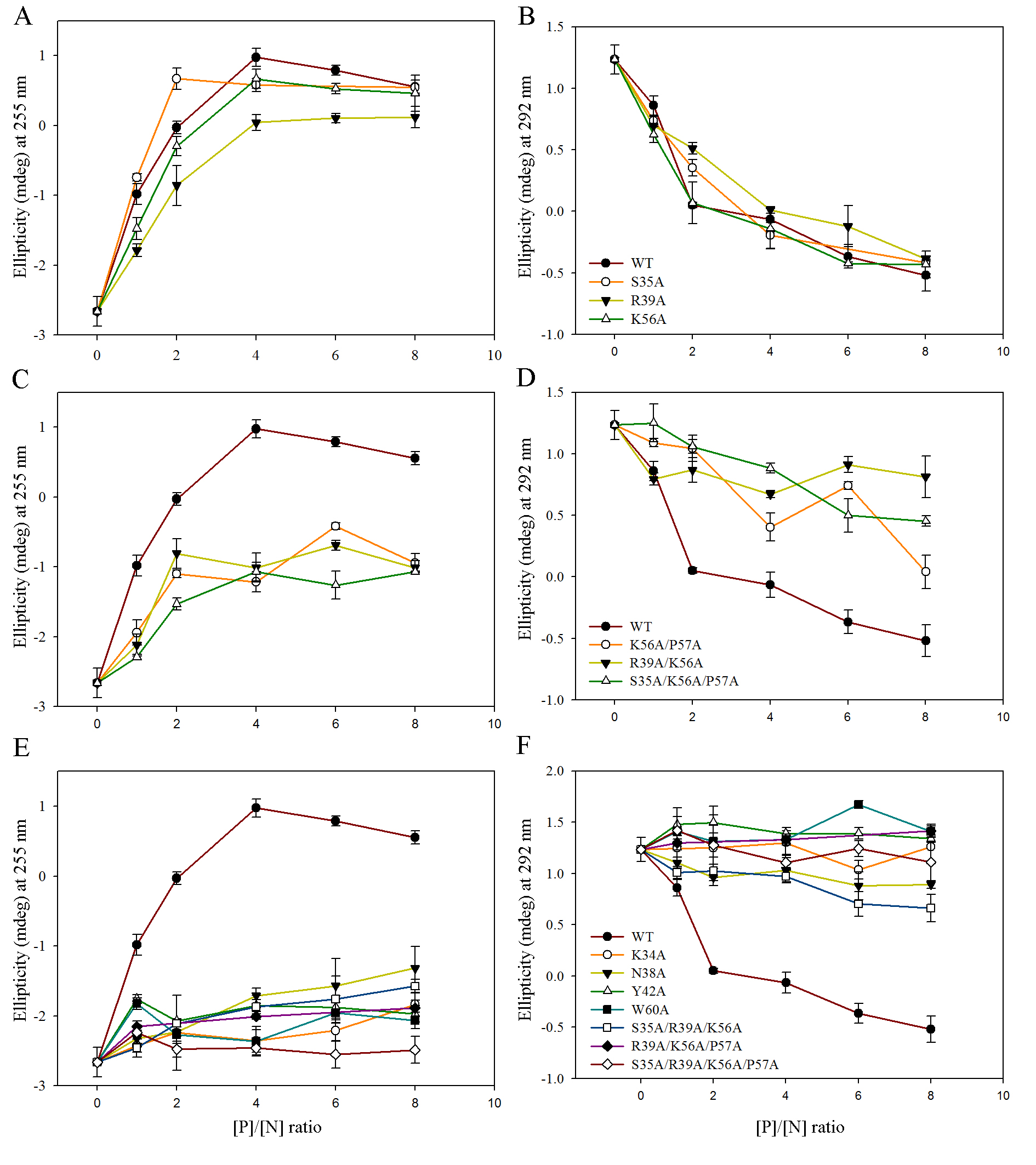


**Supplementary Figure S7**


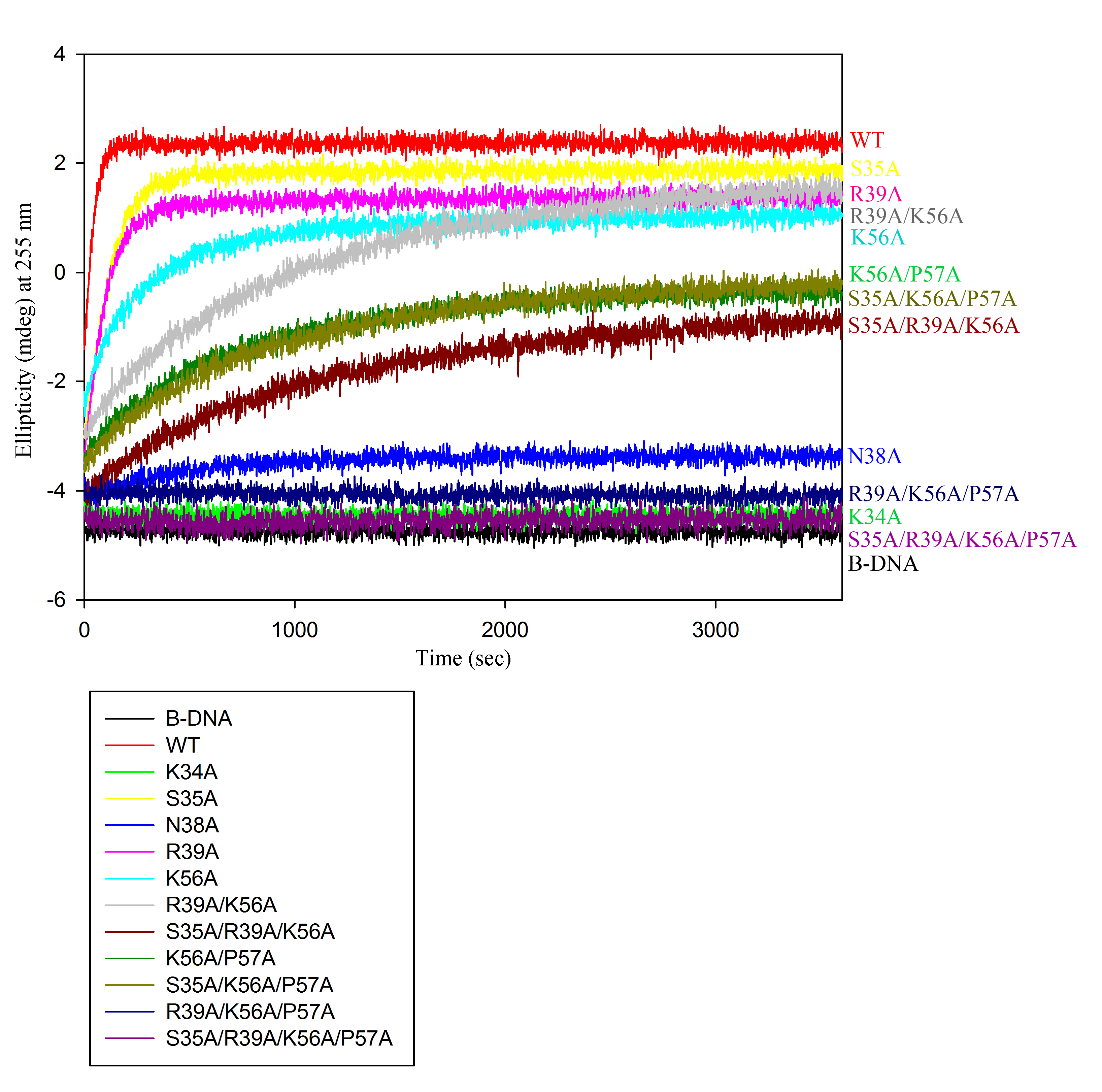


**Supplementary Figure S8**


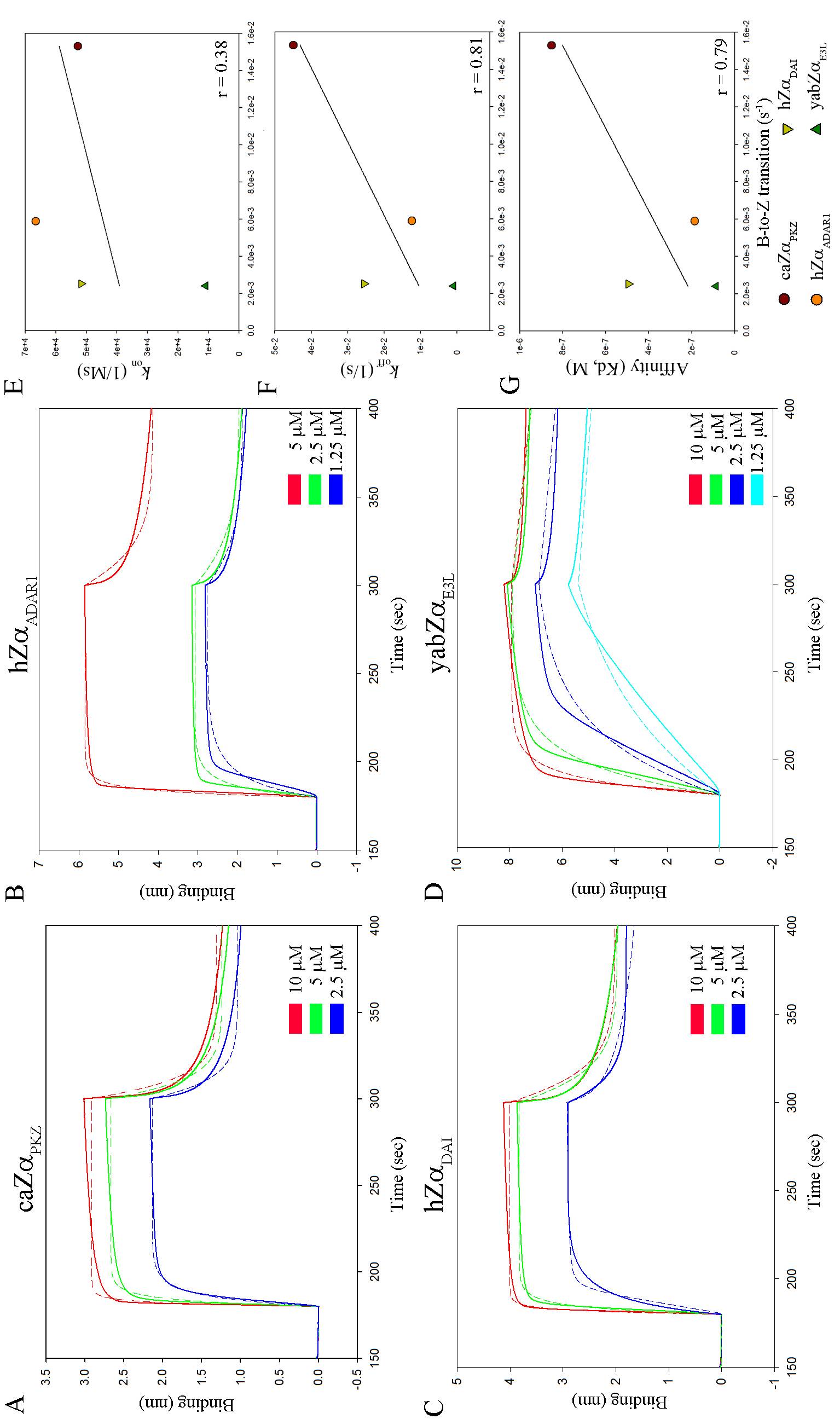


**Supplementary Figure S9**


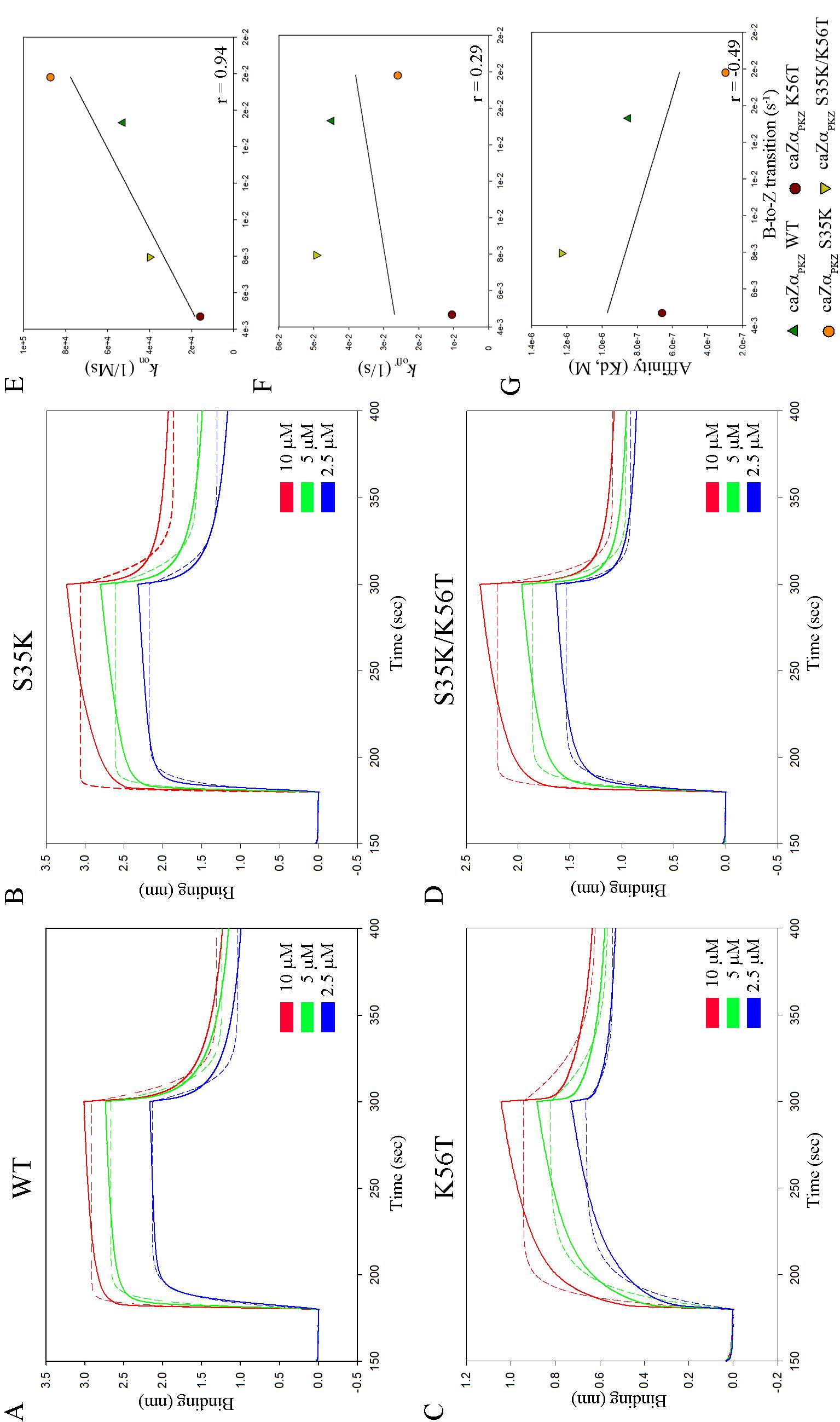


**Supplementary Figure S10**


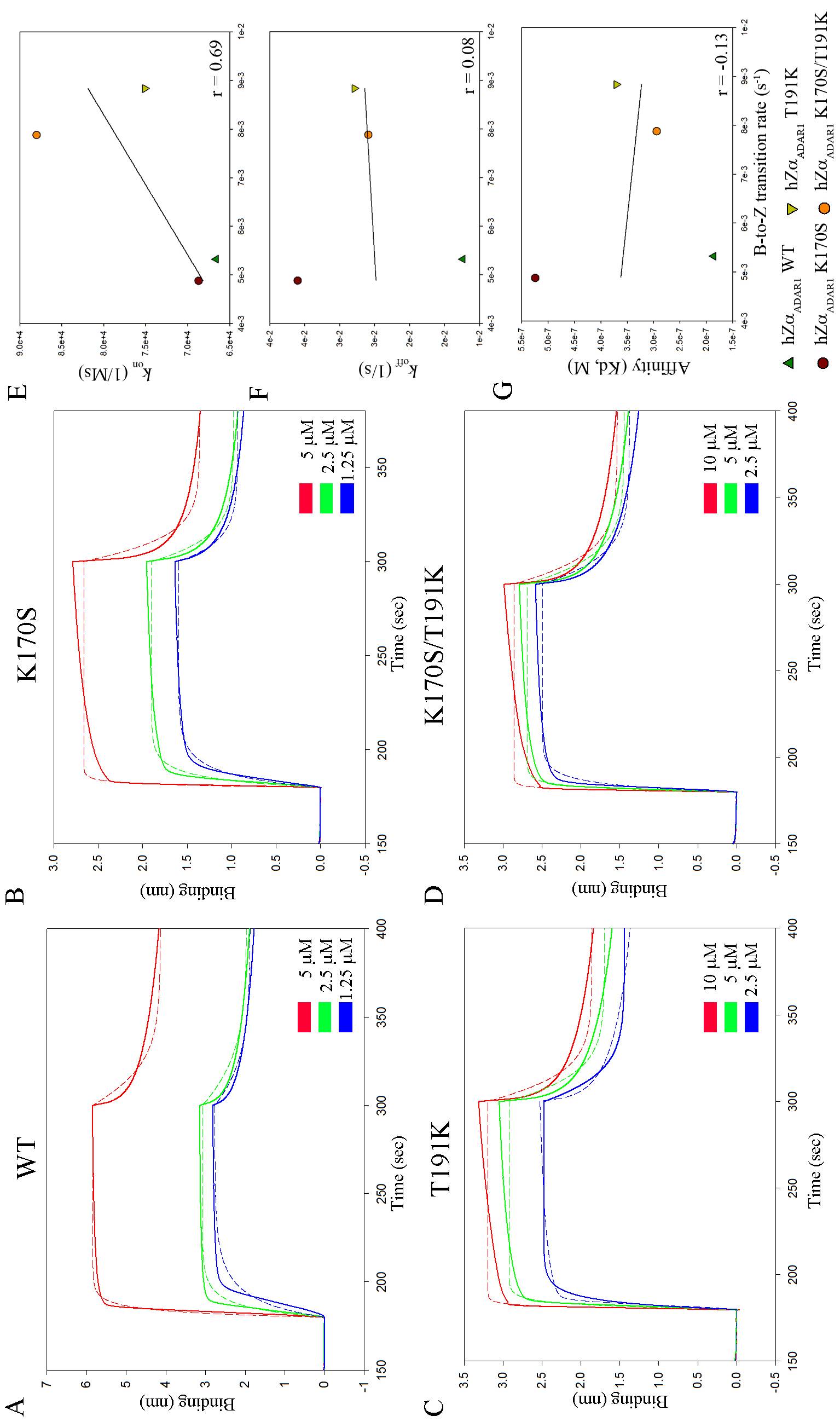


**Supplementary Figure S11**

**
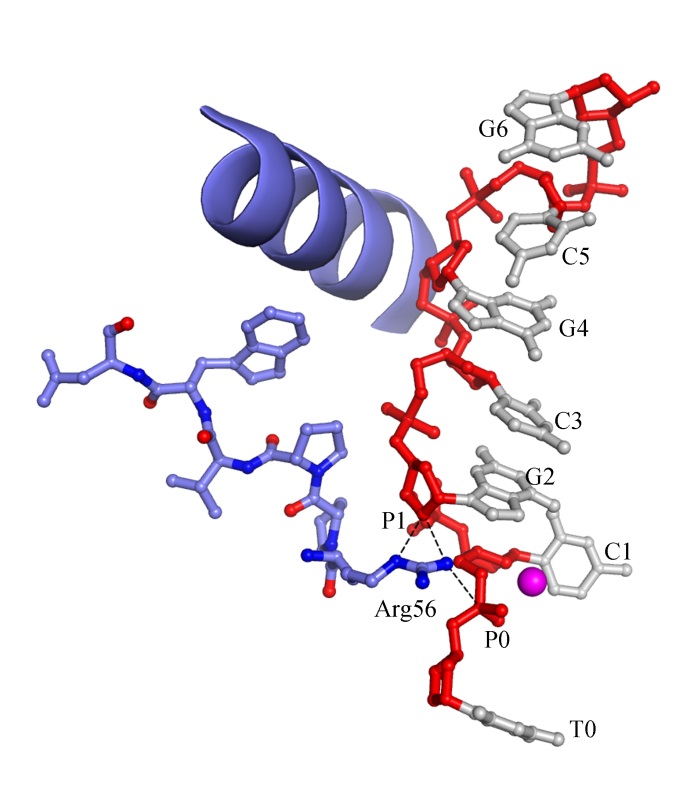
**

**Supplementary Figure S12**


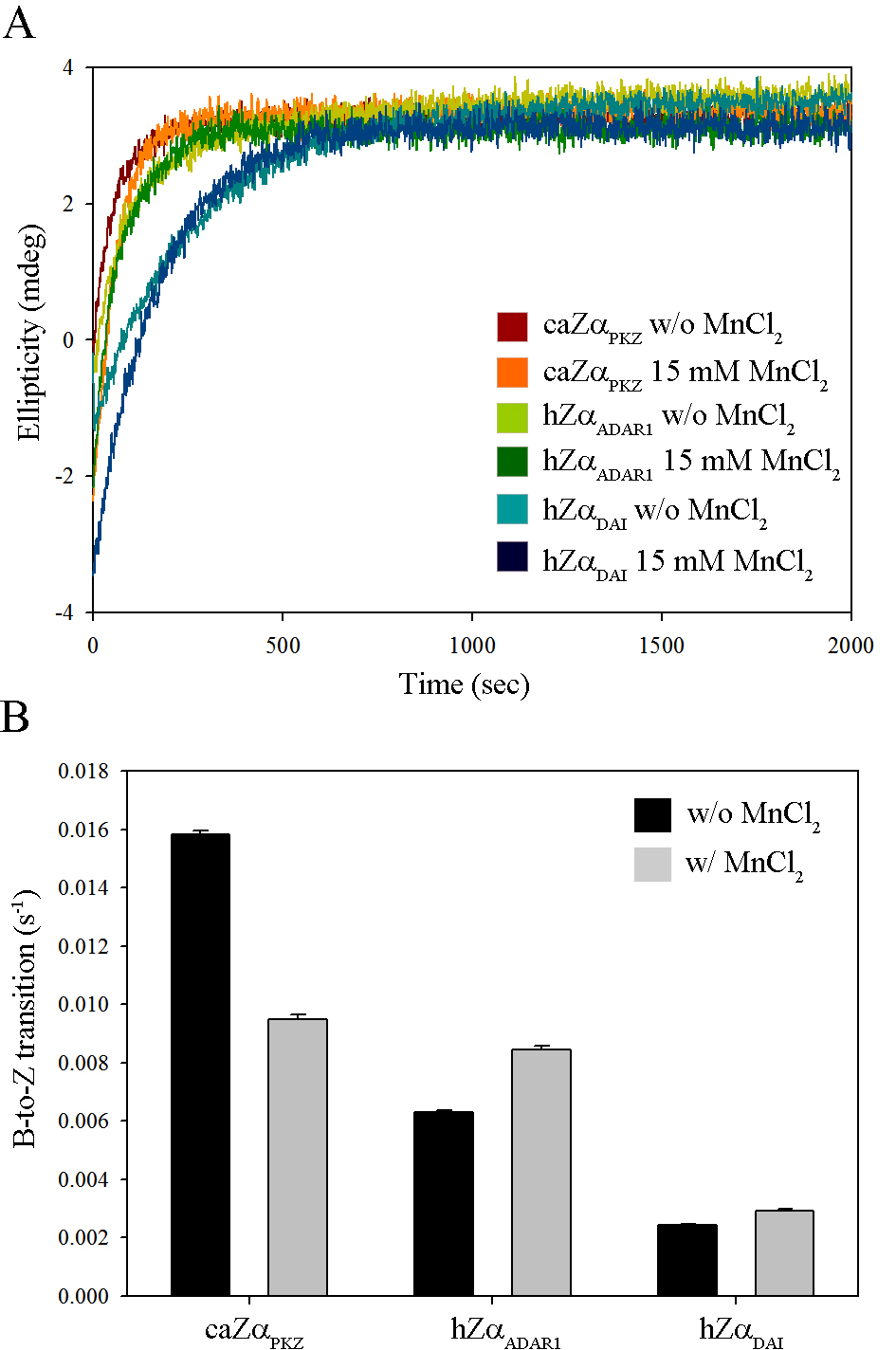

Supplement: SUPPLEMENTARY DATA [file supp_gku189_nar-01861-m-2013-File009.docx]
